# Multiple Assays on Non-Target Organisms to Determine the Risk of Acute Environmental Toxicity in Tebuconazole-Based Fungicides Widely Used in the Black Sea Coastal Area

**DOI:** 10.3390/toxics11070597

**Published:** 2023-07-07

**Authors:** Lucica Tofan, Victor Niță, Magda Nenciu, Valentina Coatu, Luminița Lazăr, Nicoleta Damir, Daniela Vasile, Dan Răzvan Popoviciu, Alina-Giorgiana Brotea, Angela Maria Curtean-Bănăduc, Sorin Avramescu, Florin Aonofriesei

**Affiliations:** 1 Department of Natural Sciences, Faculty of Natural and Agricultural Sciences, Ovidius University of Constanța, 1 University Street, 900470 Constanța, Romania; vasile_dany@yahoo.com (D.V.); dr_popoviciu@yahoo.com (D.R.P.); alina.brotea@365.univ-ovidius.ro (A.-G.B.); microbiologie_universitate_f_a@yahoo.com (F.A.); 2 Marine Living Resources Department, National Institute for Marine Research and Development “Grigore Antipa”, 300 Mamaia Blvd., 900581 Constanța, Romania; mnenciu@alpha.rmri.ro; 3 Chemical Oceanography and Marine Pollution Department, National Institute for Marine Research and Development “Grigore Antipa”, 300 Mamaia Blvd., 900581 Constanța, Romania; vcoatu@alpha.rmri.ro (V.C.); llazar@alpha.rmri.ro (L.L.); ndamir@alpha.rmri.ro (N.D.); 4 Faculty of Sciences, The “Lucian Blaga” University of Sibiu, 31 Victoriei Blvd., 550024 Sibiu, Romania; angela.banaduc@ulbsibiu.ro; 5 Department of Inorganic Chemistry, Organic Chemistry, Biochemistry and Catalysis, Faculty of Chemistry, University of Bucharest, 90 Șoseaua Panduri, 050663 Bucharest, Romania; sorin_avramescu@yahoo.com; 6 PROTMED Research Centre, University of Bucharest, 91–95 Splaiul Independenței, 050095 Bucharest, Romania

**Keywords:** fungicides, Tebuconazole, Toxkit microbiotests, acute toxicity, Gram-positive and Gram-negative bacteria, environmental risk, acute semi static, pelagic fish

## Abstract

The widespread use of Tebuconazole-based fungicides in phytosanitary treatments on a wide range of crops, on the one hand, and the lack of official reports on the amount of fungicide residues in nearby water basins, on the other hand, may lead to uncontrolled and hazardous contamination of water sources used by the resident population, and to serious effects on the environment and public health. Our study explores the acute toxicological risk of this fungicide on various organisms, from bacteria and yeast to fish, using a battery of tests (standardized Toxkit microbiotests and acute semi-static tests). By investigating the interaction between Tebuconazole and bacteria and yeast organisms, we observed that Gram-negative bacteria displayed a strong tolerance for Tebuconazole, while Gram-positive bacteria and yeasts proved to be very sensitive. The fish experiment was conducted on *Chelon auratus* juveniles exposed to five concentrations of the fungicide Tebustar EW (Tebuconazole, 250 g/L as active substance). After 96 h of exposure, the LC_50_ for *C. auratus* was 1.13 mg/L. In the case of the Toxkit microbiotests’ application, the following results were recorded: *Spirodela polyrhiza* EC_50_ = 2.204 mg/L (after 72 h exposure), *Thamnocephalus platyurus* EC_50_ = 0.115 mg/L (after 24 h), and *Daphnia magna* EC_50_ = 2.37 mg/L (after 24–48 h). With the exception of bacteria and yeast, the same response pattern was observed for all non-target species tested; the response range expressed by concentrations causing growth inhibition or mortality was small, ranging between very close values that are quite low, thereby demonstrating the high toxicity of Tebuconazole-based fungicides to the environment.

## 1. Introduction

The current population crisis is leading to increased agricultural production, and therefore to increased pesticide use, resulting in the pollution of water basins and the risk of toxicity to non-target organisms. Pesticide pollution of water resources can have serious effects on aquatic organisms and human populations; therefore, knowledge of the mechanisms of action of toxic compounds and assessment of acute toxicity to non-target organisms are essential measures to be taken for controlling and preventing pollution. In France, for example, contamination of drinking water resources with fungicides and their metabolites has recently been reported, and may lead to cancer in the human population [1].

The Tebuconazole fungicide (TEB) (active substance of Tebustar EW) belongs to the group of triazole pesticides, which have been found be toxic to non-target terrestrial plants, amphibians, terrestrial invertebrates, freshwater and marine/estuarine invertebrates, freshwater plants, and marine algae. Experiments regarding the degradation of triazole pesticides in the environment [2] have shown that epoxiconazole, Tebuconazole, and flutriafol all have a long residual period in water and soil, which calls for more attention to their pollution of the environment, and monitoring of their application to and residues in the environment. 

The product tested in our study is Tebustar EW, a fungicide for disease control in many plants such as wheat, barley, rapeseed, vines, tomatoes, and fruit trees. It is produced by the Nufarm Company, France, which has an accredited distributor in Romania and collaborations with many sales partners. The active substance of Tebustar EW is Tebuconazole—250 g/L, which is found in a proportion of 25–27%, and the adjuvant is Decanamide, which has a share of 60–70% [3]. When preparing the series of test solutions, we used the concentration value of Tebuconazole in Tebustar EW specified by the producer (250 g/L), so the results obtained in this study are expressed in mg/L Tebuconazole (active substance).

According to an EFSA Scientific Report (2008) [4] regarding pesticide risk assessment of the active substance, Tebuconazole belongs to the class of conazole fungicides alternatively classified as N-substituted triazole fungicides. Tebuconazole is the ISO common name for (RS)-1-p-chlorophenyl-4,4-dimethyl-3-(1H-1,2,4-triazol-1-ylmethyl)pentan-3-ol.

It is a systemic fungicide that penetrates into plant tissues, and active concentrations of this compound are translocated acropetally. It acts via inhibition of the demethylation at the C_14_ position in fungal sterol biosynthesis. The representative formulated products for evaluation were “Folicur EW 250”, an emulsion (oil in water) (EW) containing 250 g/L Tebuconazole, registered under different trade names in Europe, such as Tebustar EW, which is distributed in Romania by SOLAREX.SRL [4]. In the same report, the lowest endpoints driving the aquatic risk assessment were observed in tests with invertebrates and fish, such as *Mysidopsis bahia* (EC_50_ = 0.46 mg a.s./L) and *Oncorhynchus mykiss* (rainbow trout) (NOEC: 0.012 mg a.s./L) [4]. 

Tebuconazole was suspected to have endocrine-disrupting properties. A recent study was published [5], demonstrating the mechanism of adverse effects caused by Tebuconazole (TEB) on the reproduction of aquatic organisms. Four-month-old zebrafish were exposed to TEB (0.4 mg/L, 0.8 mg/L, and 1.6 mg/L) for 21 days. The study showed that TEB affected the egg production and fertilization rate by interfering with gonadal development, sex hormone secretion, and social behavior. This study provides a new perspective for understanding the mechanism of TEB-induced reproductive toxicity.

Tebuconazole has an antifungal activity, and is widely used in agriculture for the protection of crops. According to the United States Environmental Protection Agency, in terms of acute toxicity, Tebuconazole is moderately toxic to both cold-water and warm-water organisms, and highly toxic to estuarine/marine organisms [6].

Tebuconazole has a groundwater ubiquity score (GUS) index of 2.3, which means it is considered to have moderate potential for leaching into groundwater [7]. It is a non-volatile substance with a low water solubility (36 mg/L at 20 °C, purity 99.5%, pH 7.2), with aqueous hydrolysis being stable at pH 4, 7 and 9 at 25 °C, in darkness, half-life > 1 year. The time of biodegradation in aqueous photolysis (DT_50_) is 198 days for freshwater; there are no data for seawater [8]. 

Tebuconazole exhibits moderate to very high persistence in water and sediment due to its stability toward hydrolytic, photolytic, and biological degradation [9].

The use of Tebuconazole is approved in Romania, according to European Commission, under Regulation (EC) No. 1107/2009 [10].

The most recent European Decision no. 2022/1307/EU, establishing a watch list of substances for Union-wide monitoring in the field of water policy pursuant to Directive 2008/105/EC of the European Parliament and of the Council, updated the previous regulations, and several compounds such as azoles fungicides (e.g., including Tebuconazole) were included on the watch list [11]. The substances in the watch list are to be selected from those for which the information available indicates that they may pose a significant risk at Union level (to or via the aquatic environment), but for which monitoring data are insufficient to come to a conclusion on the actual risk posed. Taking this into account, the necessity of more studies on Tebuconazole in both freshwater and marine organisms arises.

Concentrations of 3.3 to 11.2 ng/L of Tebuconazole were recorded in the North-Western Black Sea Region (Dniester River Basin) according to Chitescu et al., 2021 [12]. 

Tebuconazole is moderately toxic to freshwater fishes, and LC_50_–96 h values range from 2.3 to 498 mg/L [4]. Although there are several studies about TEB’s toxicities throughout the scientific literature (especially on freshwater fish and aquatic invertebrates), there is a lack of knowledge about its impact on marine fish. A widely distributed fish species in the coastal waters of the Black Sea is the golden grey mullet *Chelon auratus* (Risso, 1810), which feeds on benthic organisms and detritus; thus, it ia continuously exposed to various xenobiotics including pesticides.

The study of Golshani et al. 2020 [13], regarding the distribution and accumulation of pesticides in three different fish species (*Liza aurata* (*Chelon auratus*), *Rutilus kutum*, and *Cyprinus carpio*) showed that the highest concentrations of pesticides were absorbed by detritivores fish such as *C. auratus*, compared to herbivorous fish (*C. carpio*) and carnivore fish (*R. kutum*). 

The literature has shown for other coastal marine species such as bivalves (see, for example, *Mytilus galloprovincialis* Lamarck, 1819) that Tebuconazole can affect both hematological parameters (hemocyte viability)and oxidative stress enzymes, and can induce histopathological changes in gills and digestive glands [14]. In fish, Tebuconazole is known to induce physiological changes in freshwater species [15], but there is a lack of data for marine species.

The purpose of this research was to highlight the effects induced by Tebuconazole on non-target organisms by evaluating its acute toxicity on both freshwater plants (*Spirodela polyrhiza*), freshwater invertebrates (*Daphnia magna*, *Thamnocephalus platyurus*) and marine fish (*Chelon auratus*). Acute toxicity tests are the most commonly used indicators to evaluate the environmental effects of pesticides. Therefore, organisms belonging to the whole food chain in an ecosystem were selected: producers, such as plants (monocotyledonous *S. polyrhiza*), consumers (such as freshwater crustaceans *T. platyurus* and *D. Magna*, and marine fish such as *C. auratus*), but also decomposers (bacteria and yeasts). 

Microorganisms are essential for matter and energy flow in terrestrial and aquatic ecosystems, mainly through the degradation and recycling of organic matter [16,17]. They can signal early the presence of toxic substances released in nature through changes in the population structure that can reverberate over all the components of an ecosystem. Due to their diverse enzymatic machinery, microorganisms are key players in the decomposition and inactivation of xenobiotics, including pesticides. For this reason, knowledge about the effect of man-made toxicants on microbial communities is important for assessing their impact on the natural environment as a whole.

It is very important to determine acute toxicity and to know the lowest concentrations at which mortality occurs, in order to protect the entire food chain in the ecosystem, and protect its integrity.

## 2. Materials and Methods

### 2.1. Application of Toxkit Microbiotests

Regulatory acceptable concentrations based on ecotoxicological data obtained from acute studies with fish, invertebrates, and primary producers are assumed to be protective to all other aquatic organisms [18]. However, further studies on other non-target organisms are needed. Toxkit’s microbiotests are an alternative to the classical tests used in ecotoxicology, and can be applied for different purposes: ecological monitoring, scientific research, and practice with students [19,20]. These alternative tests can be initiated from dormant eggs or immobilized stages, which can be hatched or de-hatched on demand, and are thus independent of the supply, cultivation, and/or maintenance of live stocks of test biota [21]. Microbiotests are a useful tool in acute toxicity investigations for the pre-screening and screening of chemicals and wastes [22].

A study on the acute toxicity of nine pesticides (two herbicides, three insecticides and four fungicides) was performed, aiming to compare the application of Toxkit’s microbiotest-based technology with conventional tests (Supplementary Material S1). The outcome of the assays revealed that the sensitivity of the microbiotests was comparable to that of the conventional tests with the same or related species, with Toxkit microbiotests even being somewhat more sensitive than conventional assays. The study confirmed that because of their culture-free nature and rapid, user-friendly and space-saving character, Toxkit microbiotests could be attractive alternatives to conventional bioassays [23]. 

#### 2.1.1. Duckweed Toxkit F

A Duckweed Toxkit F [24] growth inhibition microbiotest with *Spirodela polyrhiza* was used according to its standard operating procedure and ISO 20227 [25]. The test procedure consists of 3 days of germination of the turions in a Petri dish in Steinberg medium, at 25 °C with 6000 lux continuous illumination, followed by a transfer of the germinated turions in cups of a 6 × 8 multiwell containing five toxicant concentrations prepared in Steinberg medium, and the control without toxicant. The multiwell is incubated for 72 h at 25 °C with 6000 lux continuous illumination, after which a photo is taken from the multiwell with a digital camera and transferred to a computer. The areas of the first fronds are then measured via image analysis, and the % inhibition of the size of the first fronds in the toxicant concentrations is calculated versus their size in the controls. Data treatment and calculation of the 72 h EC_50_ is performed on specific Excel sheets, and according to the Toxkit standard operating procedure [26].

The standard operating procedure steps:

- Preparation of duckweed growth and test dilution medium (Steinberg solution);
- Germination of the *S. polyrhiza* turions (incubation 72 h at 25 °C, under continuous illumination, at min. 6000 lux);
- Preparation of the toxicant dilutions;
- Filling-in of the test plate with the toxicant dilutions;
- Transfer of the germinated turions in the test cups, photography of the multiwell at the start of the toxicity test (original);
- Incubation of the test plate (72 h at 25 °C, at min. 6000 lux);
- Photography of the multiwell at the end of the toxicity test;
- Measurement of the area of the first fronds using Image J software [27];
- Determination of the test validity: the “mean growth” of the first fronds in the cups of the control column after 3 days incubation at 25 °C and under 6000 lux illumination (=the mean t 72 h–t 0 h area) must be at least 10 mm^2^.

#### 2.1.2. Daphtoxkit F Test with *Daphnia magna* Straus, 1820

The acute *Daphnia magna* toxicity test is intended for the toxicity screening of chemicals, effluents, surface waters, wastewaters, groundwaters, sediment pore waters, and elutriates. Daphtoxkit F contains all the necessary materials to perform six acute 24–48 h mobility inhibition tests with the freshwater crustacean *Daphnia magna*. The *Daphnia* immobilization test is cost-effective, culture-independent, user-friendly and highly standardized, in compliance with ISO Standard 6341 [28] and OECD Guideline 202 [29]. The standard operating procedure steps are the following:

- Preparation of standard freshwater (S.F.) used as hatching medium for the ephippia and as a dilution medium for preparation of the toxicant dilution series;
- Pre-aeration of S.F. for at least 15 min prior to its use for the hatching of the dormant eggs and for the preparation of the toxicant dilution series;
- Hatching of the ephippia in diluted S.F., 3 days prior to the start of the toxicity test, at 20–22 °C under continuous illumination of min. 6000 lux;
- Preparation of the toxicant dilution series on TEB compound, with two phases: a range-finding test (RFT) with a dilution series of 100 mg/L; 10 mg/L; 1 mg/L; 0.1 mg/L and 0.01 mg/L, and a definitive test with concentrations ranging from 8.33 mg/L (C1) to 0.83 mg/L (C5);
- Filling-in of the test plate with the toxicant (TEB) dilution series and SF for control, in four replicates (A, B, C and D) for each test variant;
- Pre-feeding of the test organisms two hours prior to the start of exposure with a suspension of *Spirulina* powder into SF;
- Transfer of the neonates to the test wells. There are 5 *Daphnia* neonates in each well, with a total of 20 neonates for each test variant;
- Incubation of the test plate: the covered multiwell plate is incubated for 24 h and 48 h at 20 °C in darkness;
- Scoring of the results: after 24 h and 48 h of incubation, the plate is placed under a dissection microscope, and the dead and immobilized neonates are scored (they are considered dead if they do not show any movement during 15 s of observation);
- Estimation of the EC_50_ at 24 h and 48 h: a data treatment program based on Macro “REGTOX” (available on request from MicroBioTests Inc.) was applied using a sigmoid function with the EC_50_ calculation application [26].

#### 2.1.3. Thamnotoxkit F

Thamnotoxkit F contains all the necessary materials to perform six acute 24 h mobility inhibition tests with the freshwater crustacean *Thamnocephalus platyurus*. This cost-effective, culture-independent and highly standardized bioassay complies with ISO Standard 14380 [30].

The standard operating procedure steps are as follows:

- Preparation of standard freshwater (S.F.), according to the US EPA formula, used as a hatching medium for the cysts and for the toxicant dilution series’ preparation;
- Pre-aeration of S.F. and storage of 1 L S.F. (six bioassays of each Toxkit);
- Hatching of the cysts (in diluted S.F. for 20–22 h at 25°C, under continuous illumination at min. 3000–4000 lux);
- The preparation of the toxicant dilution series on chemical compounds has two phases:
  ▪ a range-finding test (RFT) with a dilution series of 100 mg/L; 10 mg/L; 1 mg/L; 0.1 mg/L and 0.01 mg/L, and
  ▪ the definitive test, in which concentrations ranged from 1 mg/L (C1) to 0.03 mg/L (C5).
- Filling of the test plate with the toxicant (TEB) dilution series and SF for control, in three replicates (A, B and C) for each test variant.
- Transfer of the larvae to the test wells (10 larvae in each well, with a total of 30 larvae for each test variant).
- Incubation of the test plate: the covered multiwell plate is incubated for 24 h at 25 °C in darkness.
- Scoring of the result: under a dissection microscope, the mortality of the larvae was scored (they were considered dead if they do not show any movement during 10 s of observation).

Estimation of the LC_50_ at 24 h. A data treatment program based on Macro “REGTOX” (available on request from MicroBioTests Inc.) was applied using a sigmoid function with the LC_50_ calculation application [26].

### 2.2. Acute Toxicity Testing on Marine Fish (Chelon auratus)

#### 2.2.1. Species Selection

The species used for testing the toxicity of Tebuconazole (Tebustar EW) on marine fish was the golden grey mullet *Chelon auratus* (Risso, 1810), indicated as reference species for the Black Sea [31] (Supplementary Material S2). *C. auratus* is an inshore brackish pelagic species, which lives close to the coast, entering lagoons and even man-made harbors, sometimes even in freshwater. It has a wide distribution in the Black Sea basin [32]. Unlike other small pelagics in the area (such as sprat—*Sprattus sprattus* (Linnaeus, 1758) and anchovy—*Engraulis encrasicolus* (Linnaeus, 1758)), the golden grey mullet adapts easily to controlled laboratory conditions, and is also suitable for breeding in captivity [31]. Additionally, fish from the Mugilidae family have been used in ecotoxicity and chemical trials (pesticides included) all around the world for more than 30 years [33,34,35,36,37,38].

#### 2.2.2. Collection, Adaptation and Conditioning

The adaptation of the test fish was recorded in two stages: firstly, adaptation of wild-caught fish to the laboratory environment and, subsequently, conditioning to experimental conditions [31].

Juvenile *C. auratus* specimens were caught from the wild (inside the Tomis Marina, Constanța, Romania), using a customized mesh net, transferred to 40-L polypropylene barrels with aeration, and transported immediately to NIMRD’s aquaculture laboratory. The fish were placed in 900-L fiberglass-reinforced plastic (FRP) tanks using a flow-through system. Water temperature was equalized by mixing small amounts of water into transportation containers until the temperature between the two containers fell within the 3 °C allowed difference [31]. 

The mean length of the fish upon collection (October 2022) was 1.95 ± 0.71 cm, with a mean biomass of 0.65 ± 0.22 g, and aged between 5–7 months. The adaptation period to laboratory conditions should not be shorter than 14 days, to allow the fish to settle and reach mortalities of the whole batch no higher than 5% [31]. In this particular case, the fish batch was kept in the laboratory for several months, recording overall mortalities below 1%. After several days of adaptation, feeding of the *C. auratus* juveniles with JBL NovoGranoColor mini pellets (2% of fish biomass daily) started.

During the entire adaptation period, the water was UV-sterilized and proper aeration was provided, ensuring an 80–90% dissolved oxygen (DO) saturation.

The second stage of the adaptation procedure (conditioning) was the process in which test fish were acclimated from standard laboratory conditions to desired experimental conditions [31]. Prior to initiating the ecotoxicity testing, 120 fish were randomly extracted from the tank and placed in a 50-L aquarium fitted with an aeration pump. The experimental batch of test fish were progressively adapted to test conditions by modifying the controlled room temperature to reach the desired testing temperature (20 ± 1 °C). The feeding was completely stopped 48 h before initiating the experiment.

#### 2.2.3. Preparation of Dilution Water

The dilution water used was natural Black Sea water pumped from a pollution-free location off NIMRD’s premises and stored in an underground decanter. Before the experiment, the water batch was analyzed; temperature, pH, salinity and dissolved oxygen were measured using a Mettler Toledo Seven Excellence multiparameter probe, while nutrients and pollutants (hydrocarbons and persistent organic pollutants) were determined by applying internationally agreed seawater analysis methods [39,40]. In order to make sure that the dissolved oxygen saturation in the dilution water was between 90% and 100% prior to use for testing, DO was measured before initiating the procedure, and when necessary, additional aeration was provided for 10–15 min. The water storage tanks were sealed and stored in the temperature-controlled room at testing temperature (20 ± 1 °C), and used the following day for Tebustar EW dilution preparation.

#### 2.2.4. Fish Randomization

In order to obtain homogeneous treatment groups and reduce potential biased judgements, fish randomization was performed following a block scheme obtained using Random Lists software [41], both for the initial distribution of fish in plastic buckets and the positioning of the test jars on the rack. The equipment and materials used during the transfer of test organisms from holding containers to test aquaria included labelled 5-L plastic buckets filled with 2 L of seawater, a small net for extracting the fish, and labelled experimental 5-L glass jars fitted with a lid. Fish were individually extracted from the conditioning aquarium using the fish net and placed one by one in the plastic buckets, carefully following the design of the randomization scheme, until the desired number of individuals was reached (four fish per replicate). Subsequently, the contents of each bucket were strained through a net, and all fish were transferred to the matching labelled experimental jar in order to avoid uneven exposure times. Finally, the jars were placed on the experimental rack, after a careful visual inspection and confirmation of conformity with the protocol (number of fish/jar) [32].

#### 2.2.5. Experimental Design of the Acute Toxicity Test

Acute toxicity testing was performed using 5-L glass jars containing a total volume of 5 L of solution. Each jar was covered with a glass lid in order to avoid any potential contamination, evaporation and/or escape of the test organisms. During the entire experimental period (96 h), gentle aeration was provided in the jars in order to maintain a DO level higher than 60%. After setting up the jars according to the randomization scheme, light intensity was measured using a Delta OHM HD 2302.0 light meter and adjusted between 540 to 1080 lx, as indicated by the US EPA guidelines [42]. Fish were exposed to the Tebustar EW solution at five dilutions: 0.5 mg/L, 1 mg/L, 2 mg/L, 3 mg/L and 5 mg/L, respectively. One control concentration (0.0 mg/L) was also used. Natural seawater was used both for control and dilution water as well. Three replicates were used for each treatment.

The Tebustar EW stock solution was prepared on the same day of testing by adding 0.25 g of Tebustar EW to seawater to volume in a 1-L calibrated glass flask and mixing on a stirrer plate for 15 min at 700 rpm. The test substance dilutions were prepared via dilution of the Tebustar EW stock solution with natural seawater in 25 L marked glass carboys, and mixed on a stirrer plate for about 15 min at 400 rpm. These volumes were then split into three replicates of 5 L in the experimental jars. Test solutions for renewal were produced in the same manner. In order to avoid excess loading with excretion products (mainly NH_4_) from the exposed fish, which may have influenced the results of the test, every 24 h, 80% of the tested solution was renewed using freshly prepared stock and dilution solutions. Renewal occurred through siphoning and replacing 80% exposure solutions from each replicate jar, as per the established protocol used [31,42]. 

Temperature, pH, salinity, dissolved oxygen, and inorganic nitrogen were measured at the beginning of the test, before the renewal (new medium), and at the end of each 24 h exposure period (old medium) for each replicate and concentration, using a Mettler Toledo Seven Excellence multiparameter probe and internationally agreed seawater analysis methods [39,40]. Nutrients dissolved in seawater (nitrites—NO_2_ and ammonium—NH_4_) were analyzed in NIMRD’s laboratory after sampling according to Grasshoff et al., 1999 [39]. Nitrates (NO_3_) were reduced to nitrites (NO_2_) with hydrazine [43] and then analyzed according to Grasshoff, 1999 [39]. A Shimadzu UV-VIS spectrophotometer was used.

#### 2.2.6. Mortality Observation and Euthanasia

Fish mortality and immobilization (an absence of swimming ability) observations were performed and recorded at 24 ± 1 h intervals after the start of the test. All dead specimens were removed. Upon completion of the 96 h, the surviving fish were euthanized humanely, according to applicable AVMA guidelines [44].

#### 2.2.7. Statistical Analysis and Interpretation

For each concentration, data from each of the three replicates were collected and the mortality rate was calculated after 24 h, 48 h, 72 h, and 96 h relative to the total number of fish used. The LC_50_ at 96 h was calculated using a sigmoid function with the LC_50_ calculation application from AAT Bioquest [45].

### 2.3. Experiments on Bacteria and Yeasts

The impact of Tebuconazole on microbial growth and viability was estimated against five bacteria and yeast strains (Table 1) (Supplementary Material S3).

#### 2.3.1. Diffusimetric Test Assay

To test the effect of Tebuconazole, we used a slightly modified well diffusimetric method [46]. The bacterial and *Candida* strains were grown overnight in Mueller–Hinton broth (MHB) and Muller–Hinton agar (MHA), respectively. Overnight bacterial and *Candida* cultures were diluted to achieve a density equivalent to 0.5 McFarland, and then used to inoculate MHA and Sabouraud Dextrose agar (SDA) plates. After inoculation, wells were cut on agar plates with sterile glass tubes (d = 6 mm). Two-fold dilutions of Tebuconazole were prepared in sterile saline water, and 100 µL of the dilutions were transferred into wells. All strains were inoculated in triplicate for each dilution and incubated at 37 °C (bacteria) and 35 °C (*Candida*), respectively. Inhibition zones were measured after 48 h, and the mean value was recorded for each dilution and strain.

#### 2.3.2. Minimal Inhibitory Concentration (MIC) Assay

MIC was evaluated using the guidelines of the Clinical and Laboratory Standards Institute (CLSI) [47]. Two-fold dilutions of Tebuconazole were prepared in MHB (bacteria) and Sabouraud Dextrose broth (SDB) (*Candida*). Subsequently, overnight cultures of bacteria and *Candida* were inoculated in test tubes to reach a final density of 1–3 × 10^6^ cell/mL. Inoculated tubes were incubated at 37 °C and 35 °C and growth was checked at 24 h and again at 48 h. The MIC was determined via visual examination of turbidity as the highest concentration of Tebuconazole without visible growth. Since dilutions with high concentrations of Tebuconazole were cloudy and prevented the accurate inspection of active culture, 2, 3, 5-triphenil-tetrazolium chloride (TTC) was used to detect growth. Some 10 µL of TTC (5%) and 10 µL of glucose (10%) were added to each test tube. After 4 h incubation, the appearance of a red colour as a result of TTC reduction indicated active growth and the absence of an inhibitory effect.

#### 2.3.3. Time-Kill Assay

The test was performed by following a modified protocol from Koeth (2022) [48]. The controls and experimental variants were prepared using Ringer solution (g/500 mL: NaCl, 1.125; KCl, 0.0525; CaCl_2_, 0.03; NaHCO_3_, 0.025, deionized water, 500 mL, pH = 6.8–7.2—Merck). The experimental variants contained concentrations of Tebuconazole both at MIC point and higher, and lower concentrations than the MIC values specific to each strain. Then, the tubes were inoculated with diluted overnight cultures to reach 1–3 × 10^6^ cells/mL. After inoculation, test tubes containing Tebuconazole, Ringer and microbial suspensions were incubated at 37 °C and 35 °C, respectively. At different time intervals (0, 2, 4, and 8 h) 100 µL were extracted, diluted and then plated out on MHA and SDA, respectively. The inoculated plates were incubated, the number of colonies developed for each variant was counted, and the mean value was plotted against time.

### 2.4. Tebuconazole Detection in the Environment

In order to determine whether or not Tebuconazole is present in the environment in the Constanţa area, water samples were collected from the Tăbăcărie and Siutghiol lakes (Supplementary Material S4).

For pesticide determination, methanol and acetone of GC-MS grade were purchased from Merck (Darmstadt, Germany). Ultra-pure water was prepared using a Milli-Q water purification system. The prepared stock solution (100 mg/L) was prepared by dissolving the standard compound in acetone, before being stored in a refrigerator at 4 °C and used for preparing six standards (5, 2.5, 0.62, 0.31, 0.07 and 0.01) mg/L.

Tebuconazole analysis was performed using a gas chromatograph (Varian 3800) equipped with a He PDD (helium pulse discharge detector) (VICI) and a CP-SIL 5CB capillary column with low bleed (packed with 100% dimethylpolysiloxane, dimension: 30 m × 0.32 mm I.D., 0.4 micron film thickness). The instrument was controlled using GC Work station software for data integration. The optimum temperature parameters were as follows: in the column oven, 60 to 270 °C at a rate of 15 °C/min., 250 °C in the injector port, and 300 °C in the detector. The injection volume was 1 µL in the split mode (split ratio 10:1), as carrier gas Helium 6.0 was used at a 1.0 mL/min. flow rate. Under these optimum conditions, a good linear relationship (detector response concentration) was observed.

For the solid phase extraction (SPE) procedure, Strata C18-E cartridges were used (C18 adsorbent, 200 mg, 3 mL, purchased from Phenomenex (Torrance, CA, USA). SPE columns were previously conditioned using 3 mL of methanol and 3 mL of purified water. After the conditioning phase, 250 mL of water samples (sampled from different locations and previously filtered on paper filter) were introduced into the SPE columns at a flow rate of 6 mL/min. In the same conditions, distilled water samples were fortified by the addition of a volume of stock solution corresponding to 5 mg/L. Finally, the analytes were eluted with 1 mL of acetone, and the obtained extract was injected into the chromatographic system. 

## 3. Results and Discussion

### 3.1. Results of Toxkit Microbiotests

A battery of micro bioassays should in principle be composed of representatives of the producers (algae, higher plants), consumers (mostly zooplankton or zooedaphon invertebrates), and decomposers (bacteria or fungi) [49]. 

#### 3.1.1. *Spirodela polyrhiza*

The percentual inhibition of frond area growth in *Spirodela polyrhiza* plants (Figures S4 and S5 in Supplementary Material S1) varied from 15.67% (at 0.39 mg/L TEB) to 91.65% (at 6.25 mg/L TEB). 

**Figure 1:**
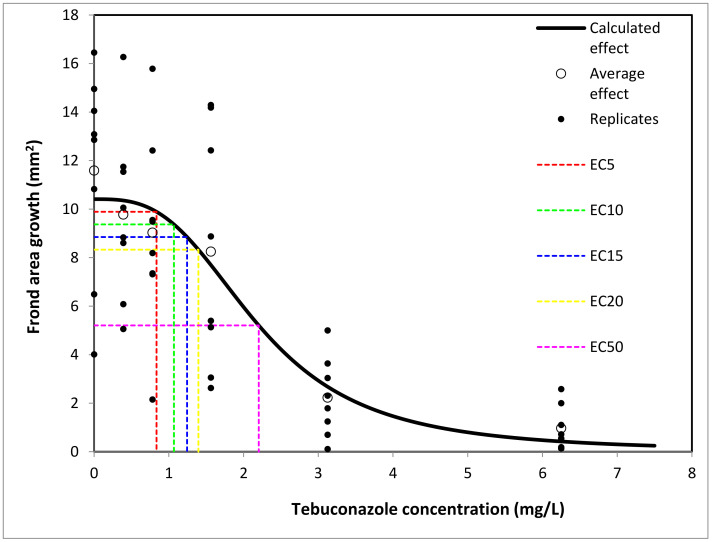
Frond area of *Spirodela polyrhiza* plants after 72 h of exposure to Tebuconazole. ECx (x = 5, 10, 15, 20 and 50) values are represented.

The 72 h EC_50_ for *S. polyrhiza* was 2.204 mg/L (Table 2, Figure 1).

According to the producer-provided security data sheet, the 72 h EC_50_ for the related water plant *Lemna gibba* is reported to be 0.144 mg/L [3], which is a significantly lower value than that found for *Spirodela polyrhiza*. Tests on *Lemna gibba* found an EC_50_ for plant growth of 0.144 mg/L after 14 days of exposure (and 0.180 for biomass growth inhibition) according to the European Food Safety Authority [9], and 0.151 mg/L according to the US Environmental Protection Agency [50]. A value of 0.44 mg/L, also lower than the 72 h EC_50_ for Tebuconazole, was found to be the 72 h EC_50_ in *Spirodela* for a related fungicide, econazole [51]. Similarly, prothioconazole and prothioconazole-desthio (a metabolite formed following its degradation) had a variable effect on *Lemna minor*, depending on the enantiomer used, with a 72 h EC_50_ of 7.59–20.36 mg/L (and 0.76–5.63 at 7 days). The main consequences of prothioconazole exposure seem to be an inhibition of photosynthetic pigments’ synthesis, malonildialdehyde accumulation, and increased oxidative stress [52]. 

On the same *Lemna minor*, the average growth rates EC_50_ after 7 days of exposure were found to be 1.851 mg/L for fluconazole and 1.069 for propiconazole, respectively [53]. Ketoconazole was shown to be even more toxic, with an EC_50_ after 7 days of 0.08–0.16 mg/L, and effects including root and frond growth inhibition and lower biomass [54].

Difeconazole is also toxic to *L. minor*, affecting frond growth and chlorophyll levels above a 0.5 mg/L concentration [55]. 

In another duckweed species, *Landoltia punctata*, the herbicide uniconazole was shown to inhibit amylaze enzymes and alter hormone synthesis, leading to abnormally high starch levels [56]. 

Other conazole pesticides found to be toxic to aquatic angiosperms are epoxiconazole and paclobutrazole, with high toxicity on *Lemna gibba* [57].

Comparison with these results shows that Tebuconazole is highly toxic, but not among the most commonly known conazoles that are toxic to Lemnoideae. The use of standardized micro bioassay toxicity tests was shown to be a reliable option in the evaluation of the toxicity of an insecticide to non-target organisms, demonstrating ease, accuracy, and a short time taken for application and the evaluation of results [58].

#### 3.1.2. *Daphnia magna*

According to the producer-provided security data sheet, the 48 h EC_50_ for a conventional test with *Daphnia magna* is reported to be 2.37 mg/L for Tebuconazole and 16 mg/L for Tebustar EW. The NOEC effect for chronic exposure is 0.01 mg/L [3].

**Figure 2:**
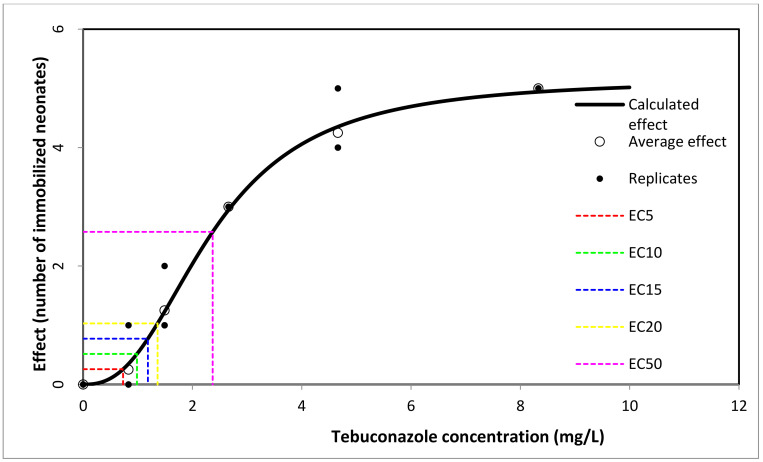
Number of immobilized *D. magna* neonates after 48 h exposure to Tebuconazole.

In our study, the lowest immobility result of *Daphnia* neonates was produced by 0.73 mg/L Tebuconazole (EC_5_ = 5% imobility of *Daphnia* neonates). The 48 h EC_50_ was 2.37 mg/L Tebuconazole (95% confidence interval: 2.266–2.521) (Figure 2, Supplementary Material S6).

According to a scientific Report of EFSA, the NOEC on the long-term time scale for Tebuconazole is 0.01 mg/L [4].

There are many studies on the acute toxicity of Tebuconazole on aquatic invertebrates, especially on *Daphnia magna* [9,59], with 48 h EC_50_ values ranging from 1.9 mg/L to 3.53 mg/L. Our result for the 48 h-EC_50_ value of 2.37 mg/L Tebuconazole is similar to that specified by the producer [3] 

The choice of the two invertebrate organisms (*D. magna* and *T. platyurus*) may be motivated by the fact that both are part of zooplankton, an important component of aquatic food webs, which are very sensitive to environmental pollution; they develop forms of resistance known as “dormant eggs”, which can be used in the development of standardized Toxkit microbiotests [21].

#### 3.1.3. *Thamnocephalus platyurus*

**Figure 3:**
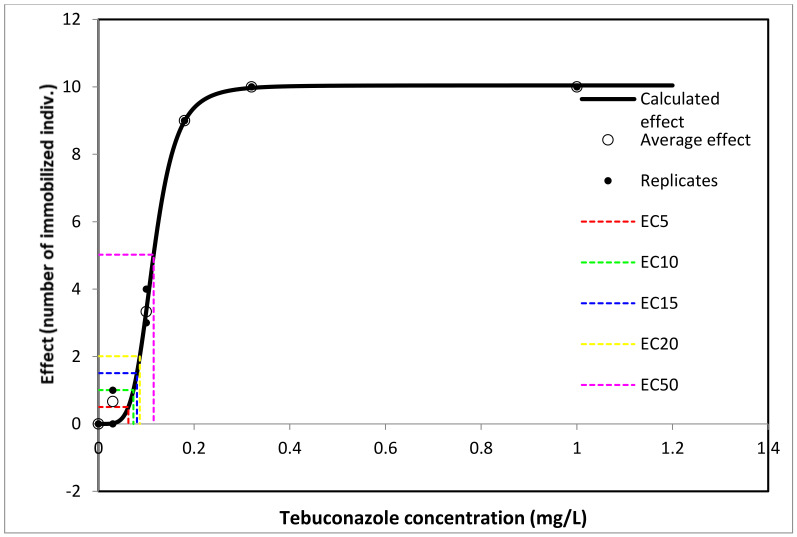
Number of immobilized *T. platyurus* individuals after 24 h of exposure to Tebuconazole.

The lowest TEB concentration with a mortality effect in *Thamnocephalus* larvae was 0.063 mg/L TEB (5% mortality), and the highest TEB concentration was 0.32 mg/L (100% mortality). The 24 h EC_50_ was 0.115 mg/L (95% confidence interval: 0.110–0.118) (Figure 3, Supplementary Material S7).

The studies made for the three trophic levels (fish, invertebrates and aquatic plants) showed that the lowest acute aquatic toxicity values for Tebuconazole recorded were 0.46 mg/L (*Mysidopsis bahia*) and 0.237 mg/L (*Lemna gibba*) in invertebrates and aquatic plants, respectively. Tebuconazole, therefore, fulfils the criteria for classification as Aquatic Acute Cat. 1. [60]. Information on its acute toxicity to other invertebrates besides *Thamnocephalus platyurus* are scarce; therefore, our results may contribute to the enrichment of knowledge about this species. Comparing the results obtained between the two species of invertebrates tested, *T. platyurus* is an order of magnitude more sensitive than *D. magna.*

### 3.2. Acute Toxicity Results on C. auratus

**Figure 4:**
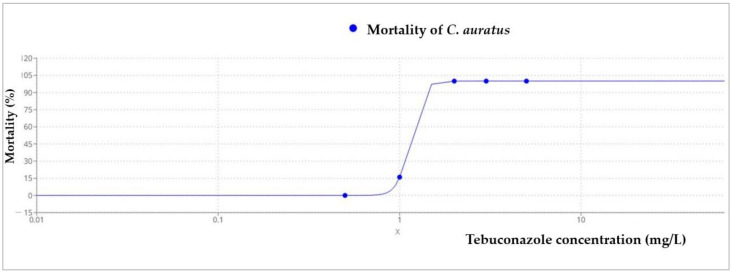
Mortality of *C. auratus* after 96 h exposure to Tebuconazole (LC_50_ = 1.13 mg/L).

All golden grey mullet individuals exposed to the highest concentration of Tebuconazole (5 mg/L) died during the first 24 h, while fish exposed to 3 mg/L died after 48 h, and those exposed to 2 mg/L died after 72 h. Overall, the LC_50_ at 96 h for Tebuconazoleon *C. auratus* juveniles was 1.13 mg/L (Figure 4, Table 3) (95% confidence interval: 0.963–1.311).

The toxicity test performed was considered valid from the fish survivability point of view, as no mortalities were recorded in the control batch [31]. 

All water quality parameters (temperature, salinity, pH, DO, nutrients) (Supplementary Material S8) varied within the range stipulated by the protocol [31]. It should be noted that if as in the case of NO_2_ and NO_3_, no significant changes were observed during the experiment, in the case of NH_4_, the values were always higher every 24 h, most likely as the result of the fish’s biological processes (the accumulation of excretion products).

While Tebuconazole was proven to be moderately toxic to freshwater fishes (LC_50_—96 h values ranging between 2.3 to 498 mg/L) [4], the LC_50_ of 1.13 recorded on *C. auratus* during this test suggests that marine species might be more sensitive. As golden grey mullets, detritivore fish, are widely distributed in coastal Black Sea waters, they are constantly exposed to the xenobiotics (fungicides included), which reach the marine environment in run-off from agriculture in the catchment area. The lack of knowledge on pesticide impact on marine fish clearly calls for a more in-depth study of their acute and chronic effects.

### 3.3. Results Obtained from Bacteria and Yeast Tests

#### 3.3.1. Diffusimetric Test

The diffusimetric tests indicated major differences in the tolerance of microbial strains to Tebuconazole. Gram-negative bacteria, especially those belonging to the genus *Pseudomonas*, showed a high level of resistance to Tebuconazole. At concentrations of 15.625 mg/mL and at concentrations lower than this, no growth inhibition was recorded. Additionally, *E. coli* showed a high tolerance to Tebuconazole (maximum inhibition zone of 4 mm), although to a somewhat lesser extent (Table 4).

A different situation was observed for *Candida* and *Bacillus*, both being inhibited by very low concentrations of Tebuconazole (0.061 mg/mL).

#### 3.3.2. Results of MIC Estimation

The MIC estimation largely confirmed the observations of the diffusimetric tests. Thus, both strains of *Pseudomonas* had an MIC value of 31.25 mg/mL, while for *E. coli*, the value was 15.62 mg/mL. These values showed the high tolerance of Gram-negative bacteria to Tebuconazole.

The MIC value was much lower for *Candida* ATCC, at only 0.0012 mg/mL, while the MIC value of *Bacillus* was very close, but also significantly low, at only 0.0024 mg/mL (Table 5). It is likely that the difference in tolerance between the two groups of bacteria can be explained by the ultrastructural characteristics of the cell. 

#### 3.3.3. Results of Time-Kill Assay

We also performed time-kill experiments to see how the viability of microorganisms is affected by exposure time to Tebuconazole, and included a wide range of Tebuconazole concentrations. The manner in which the decrease in cell viability occurred over time could provide a model for assessing Tebuconazole’s potential effect on microbial communities in the natural environment.

**Figure 5:**
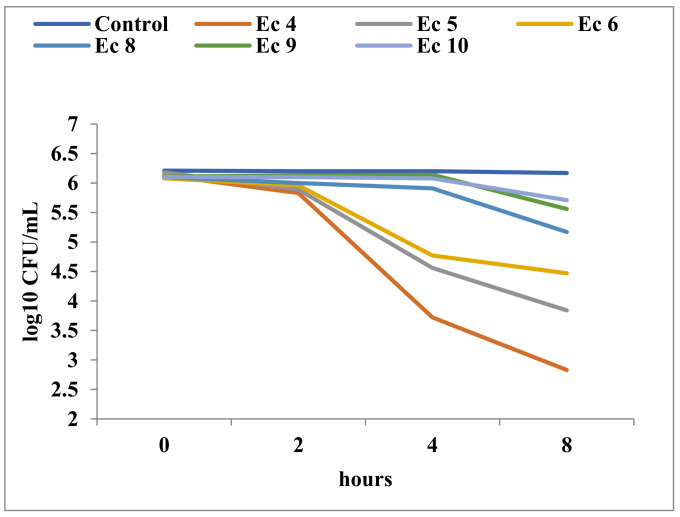
Time-kill in *E. coli* ATCC 25922 at different concentrations of Tebuconazole (Ec4 = 15.625; Ec5 = 7.81 mg/mL; Ec6 = 3.90 mg/mL; Ec8 = 0.97 mg/mL; Ec9 = 0.48 mg/mL; Ec10 = 0.24 mg/mL).

**Figure 6:**
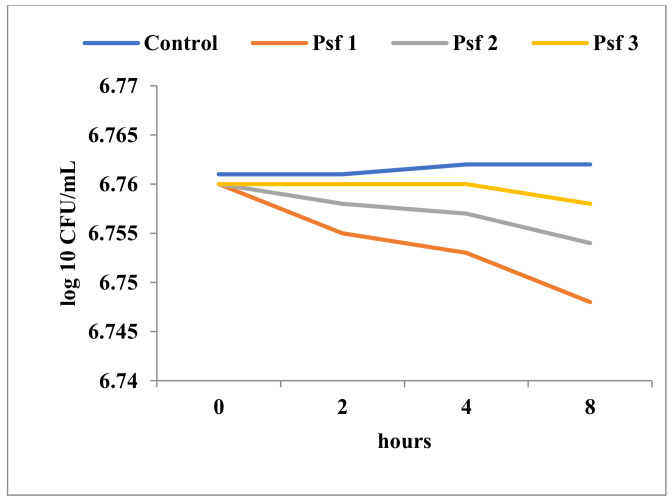
Time-kill in *P. fluorescens* at different concentrations of Tebuconazole (Psa1 = 125 mg/mL; Psa2 = 62.5 mg/mL; Psa3 = 31.25 mg/mL).

**Figure 7:**
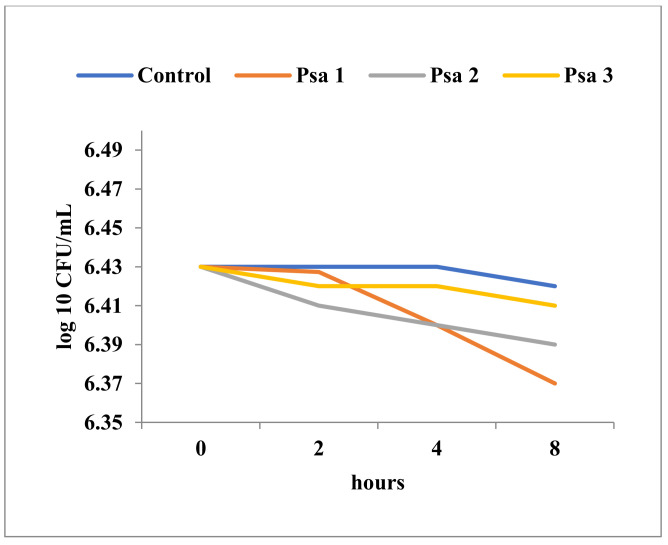
Time-kill in *P. aeruginosa* ATCC 27853 at different concentrations of Tebuconazole (Psa1 = 125 mg/mL; Psa2 = 62.5 mg/mL; Psa3 = 31.25 mg/mL).

The decrease in cell viability upon exposure to Tebuconazole was variable and determined by the affiliation to a particular taxonomic group, as well as by the ultrastructural characteristics of the respective species. As expected, Gram-negative bacteria showed minimal cell population decreases over time, even at high concentrations of Tebuconazole (Figure 5, Figure 6 and Figure 7). In general, these bacteria showed a high degree of resistance to high concentrations of Tebuconazole. In *E. coli*, the decrease in cell viability was affected to a greater extent by the concentration of Tebuconazole, and to a lesser extent by the exposure time (Figure 5).

Unlike *E. coli*, the cell viability of the two *Pseudomonas* species (Figure 6 and Figure 7) was apparently not significantly affected, even at very high concentrations of Tebuconazole (125 mg/mL). Thus, at 125 mg/mL, the number of viable cells of *P. aeruginosa* ATCC was only slightly reduced from 6.43 log10 CFU/mL to 6.37 log10 CFU/mL (Figure 7).

Somewhat surprising were the cell mortality dynamics in *Bacillus*, which were quite similar to those of *Candida*, although taxonomically and ultra-structurally, the two microorganisms are very different.

**Figure 8:**
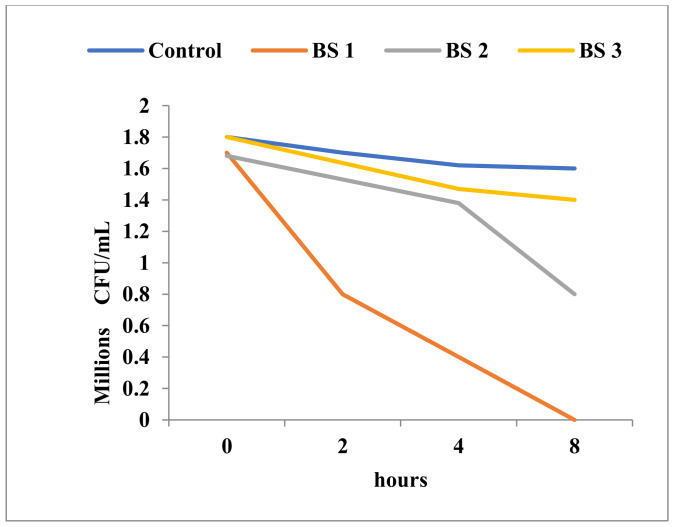
Time-kill in *Bacillus* sp. at different concentrations of Tebuconazole (BS1 = 0.25 mg/mL; BS2 0.025 mg/mL; BS3 = 0.0125 mg/mL).

**Figure 9:**
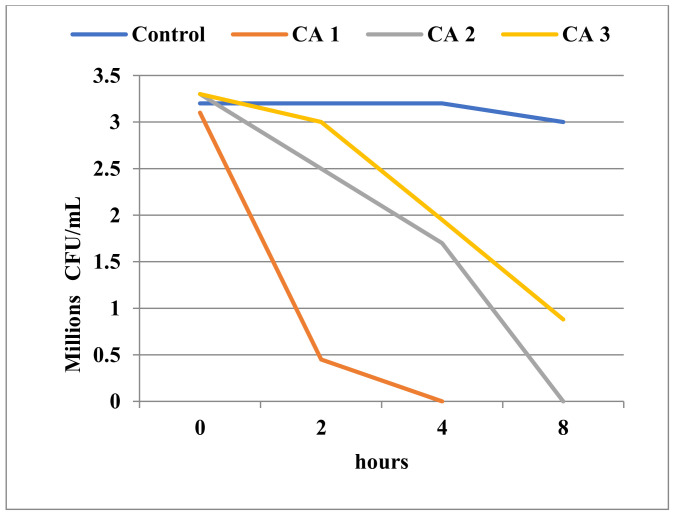
Time-kill in *Candida albicans* ATCC 10231 at different concentrations of Tebuconazole (CA1 = 0.25 mg/mL; CA2 = 0.05 mg/mL; CA3 = 0.025 mg/mL).

At 0.25 mg/mL Tebuconazole, the *Bacillus* population with an initial density of 1.6 × 10^6^ CFU/mL cells was inactivated in 8 h (Figure 8). This undoubtedly evidenced a strong toxic effect, one very similar to the activity of a true antimicrobial compound. Not surprisingly, Tebuconazole had a very intense inhibitory effect against *Candida*, and had the ability to destroy the entire initial population of about 3 × 10^6^ cells in 4 h of exposure at a concentration of 0.25 mg/mL (Figure 9).

The laboratory experiment on pure cultures showed that Tebuconazole had a toxic effect on bacteria, manifested selectively on Gram-positive bacteria, while Gram-negative bacteria could tolerate high concentrations. 

Although *Candida* is a member of the fungi group, it has no phytopathogenic significance and can therefore be included as a non-target organism. However, *Candida* shares lanosterol demethylase with the other fungi; this is the enzyme involved in the synthesis of the fungal cell membrane, and at the same time is a target for azole antifungal drugs. Tebuconazole inhibits the activity of this enzyme and causes a decrease in the amount of ergosterol in fungal membranes, which leads to the alteration of their normal function. In turn, the cells cannot carry out their normal multiplication functions, which leads to their death. This leads to the loss of the ability to divide, and the death of the cells [61,62]. Therefore, it is not at all surprising that a *Candida* cell population of 3 × 10^6^ CFU/mL can be killed in just 4 h by Tebuconazole at concentrations of 0.25 mg/mL. Since the above results are laboratory determinations, it is difficult to evaluate the potential impact of Tebuconazole on the natural microbial communities living in soil, water, or sediments. Although there are several studies regarding the fate of Tebuconazole in nature, it is not yet clear what impact this fungicide has on the function, biomass, and composition of microbial communities. There is still no clear principle applicable on a large scale to all natural environments [63,64,65]. Some studies have found insignificant effects of Tebuconazole treatment on the bacterial and fungal community structures living in sediments and leaf material [18]. Bacmaga et al. (2022) [66] found that Tebuconazole did not apparently affect the normal biological characteristics of soils after its use in agriculture. The authors showed that on the contrary, after the application of Tebuconazole, a stimulation of heterotrophic bacteria and fungi was observed, as well as the enzymatic activities of soil, such as those of dehydrogenases, alkaline phosphatase, and beta glucosidase. At the same time, they detected a change in the diversity of microbial physiological activities after the use of the fungicide. Other authors [67,68] have noted a significant decrease in microbial biomass after the use of high doses of Tebuconazole. Toxic concentrations for fish and invertebrates do not seem to have a visible effect on microbial communities associated with sediments or leaf material in microcosm systems [18,66,69,70]. The influence of Tebuconazole on aquatic microbial communities was moderate, and its effect depended on the particularities of the respective ecosystems [71,72]. Youness et al. (2018) [73] screened several bacterial strains in the laboratory for the degradation of this fungicide. Among other species, they analyzed 13 strains of *Bacillus,* and found only three capable of degrading Tebuconazole. Other reports [74] have shown that on the contrary, the growth of *B. subtilis* is inhibited by Tebuconazole; these results are similar to our observations. 

The strong and apparently selective inhibitory activity against *Bacillus* compared to Gram-negative bacteria (*E. coli*, *Pseudomonas* sp.) implies the existence of target sites for binding azoles, with molecular structure targets similar to those existing in fungi, in at least some Gram-positive bacteria.

*Bacillus* sp. synthesizes several cytochrome P450 mono-oxygenases (P450s) [75,76,77,78] closely related to eukaryotic CYP51 (cytochrome P450 family 51) [79], a target site for azole drugs [80]. This could explain the strong inhibitory effect of Tebuconazole on *Bacillus*, with values almost equal to those recorded for *Candida*. This enzyme is missing in *E. coli* and probably in many Gram-negative bacteria [80], a fact that correlates with the high resistance of this group to Tebuconazole, along with other factors.

It is very likely that microbial decomposition capacity is often limited in natural environments. The long-term use of this compound at high concentrations can lead to disruption of the composition of natural microbial communities, especially in soil environments. Changes in microbial diversity could take place in microenvironments that favour the accumulation of high concentrations of Tebuconazole. The release and accumulation of Tebuconazole in the environment could negatively affect non-pathogenic, heterotrophic yeasts and fungi, as well as a part of the Gram-positive bacterial community. However, the toxic and inhibitory effect of Tebuconazole on microbial communities in natural environments remains debatable. This is because its direct toxic effect is related to the presence of high doses, which are unusual in agricultural practice.

### 3.4. Tebuconazole Detection in the Environment

The results of pesticide detection (Table 6) indicated that Tebuconazole is indeed present in the Constanţa coastal area, as a consequence of its use in agriculture practices. The detection of Tebuconazole in the two lakes that flow into the sea, with concentrations close to the results obtained in this study, shows that there is a major risk for aquatic organisms in the coastal zone (plants, invertebrates and fish), which may lead to a decrease in biodiversity and should trigger an alarm for future investigations.

## 4. Conclusions

In all species tested with Toxkit microbiotests (*S. polyrhiza*, *D. magna* and *T. platyurus*), as well as in the case of *C. auratus*, the same response pattern was observed: the response range, as expressed by concentrations causing growth inhibition or mortality, lies between very close values which are quite low, thereby demonstrating the high toxicity of Tebuconazole-based fungicides in the environment.

Laboratory experiments showed that a direct toxic effect of Tebuconazole occurred selectively on Gram-positive bacteria, while Gram-negative bacteria could tolerate very high concentrations of Tebuconazole. Our data suggest that the use of Tebuconazole may affect the composition of natural microbial populations and cause subtle changes in their functioning, especially in terrestrial environments. This can occur following the accumulation of high doses of Tebuconazole, especially in small areas such as microenvironments. Additionally, our data suggest that the impact of Tebuconazole could be more significant on heterotrophic yeasts and fungi, as well as Gram-positive bacteria.

Acute toxicity testing on marine fish, conducted on golden grey mullet (*C. auratus*) juveniles (as a representative of pelagic species on the Romanian coast), determined a 96 h LC_50_ of 1.13 mg/L. When compared to the reported toxicity of Tebuconazole on freshwater fish (showing higher LC_50_ thresholds), this value suggests that marine species might be more sensitive. Such a finding strongly underpins the need to deepen research into both the chronic and acute effects of fungicides on marine fish, given the fact that mullets are exposed to run-off, resulting from agriculture in the Dobrogea area, which drains into the Black Sea.

We believe that our study can make an important contribution to the monitoring and prevention of pollution from pesticides used in agriculture that has been proven by the detection of the Tebuconazole-based fungicides in coastal waters at concentrations similar to those tested in the laboratory, and which may pose an ecotoxicological risk to coastal biota and human health.

## Figures and Tables

**Table 1:** The strains used to estimate the effect of Tebuconazole on microbial growth.

No.	Strain	Observation
1	*Escherichia coli* ATCC 25922	Reference strain
2	*Pseudomonas aeruginosa* ATCC 27853	Reference strain
3	*Pseudomonas fluorescens*	Clinical strain
4	*Bacillus* sp.	Gram-positive, spore-forming soil isolate
5	*Candida albicans* ATCC 10231	Reference strain

**Table 2:** Frond area growth inhibition of *Spirodela polyrhiza* plants after 72 h in Tebuconazole solutions.

Indicative	Concentration (mg/L)	Average Inhibition (%)	72 h EC_50_ (mg/L)	95% Confidence Interval
C1	6.25	91.65	2.204	1.031–2.946
C2	3.12	80.78		
C3	1.56	28.84		
C4	0.78	22.09		
C5	0.39	15.67		
M	0 (control)	-		

**Table 3:** LC_50_ regression equation results (mortality of *C. auratus*).

Parameter	Value
LC_50_	1.1372
Equation	$Y=-0.0036+\frac{100.0232+0.0036}{1+{\left(\frac{x}{1.1372}\right)}^{-12.8991}}$
Equation Form	$Y=Min+\frac{Max-Min}{1+{\left(\frac{x}{LC50}\right)}^{Hill\;coefficient}}$

**Table 4:** Inhibitory effect of Tebuconazole against the tested microbial strains.

Two-Fold Dilutions	I	II	III	IV	V	VI	VII	VIII	IX	X	XI	XII	XIII
Tebuconazole concentration mg/mL	125	62.5	31.25	15.625	7.81	3.90	1.95	0.97	0.48	0.24	0.12	0.061	0.030
*E coli* ATCC 25922	4	3	2	2	0	0	0						
*P. aeruginosa* ATCC 27853	3	2	1	0	0	0	0						
*P. fluorescens*	3	3	1	0	0	0	0						
*Candida albicans* ATCC 10231	20	20	16	12	12	11	11.5	9.5	9	7.5	6	3	0
*Bacillus* sp.	12	10	9	8	8	7	6.5	7.5	6	4	4	3.5	0

**Table 5:** MIC value of Tebuconazole against tested microbial strains.

Strain	mg/mL Tebuconazole
*E. coli* ATCC 25922	15.62
*P. aeruginosa* ATCC 27853	31.25
*P. fluorescens*	31.25
*Candida albicans* ATCC 10231	0.0012
*Bacillus* sp.	0.0024

**Table 6:** Tebuconazole values detected in water samples (Tăbăcărie and Siutghiol lakes) in March 2023.

Sampling Station	TEB (mg/L)	SD (±)
Ramada	2.02	0.04
Large bridge	1.47	0.11
On Plonge Jr.	0.5	0.06
Tăbăcărie Microreserve	0.96	0.15
Church	0.41	0.08
Sports Camp	0.38	0.05
Expo	0.27	0.036
Station 2 trees	0.084	0.009
Station 1 stairs	0.098	0.021

## Data Availability

Data is contained within the article or Supplementary Materials.

## Supplementary Materials

The following supporting information can be downloaded at: https://www.mdpi.com/article/10.3390/toxics11070597/s1, S1: Procedures of TOXKIT Microbiotests; S2: Experiments on marine fish (*Chelon auratus*); S3: Experiments on bacteria and yeasts; S4. Analysis of water samples for pesticide content detection; S5: Duckweektoxkit F *Spirodela* Regtox statistics; S6: MBT *Daphnia* Regtox statistics; S7: MBT *Thamno* Regtox statistics; S8: Water quality parameters (temperature, salinity, pH, DO, nutrients) during TEB acute toxicity testing on *C. auratus.* References [81,82,83,84,85,86,87] are cited in the Supplementary Materials.
