# Supplementary material for: Multiple Assays on Non-Target Organisms to Determine the Risk of Acute Environmental Toxicity in Tebuconazole-Based Fungicides Widely Used in the Black Sea Coastal Area
Source: Toxics. 2023 Jul 7;11(7):597. doi: 10.3390/toxics11070597 (PMC10385278; doi:10.3390/toxics11070597)
Supplement: Supplementary file 1 [file toxics-11-00597-s001.zip › S1. Procedures of TOXKIT Microbiotests.docx]

**S1. Procedures of TOXKIT Microbiotests [81]**

**DUCKWEED TOXKIT F** with *Spirodela polyrhiza* ([L.](https://en.wikipedia.org/wiki/Carl_Linnaeus)) [Schleid](https://en.wikipedia.org/wiki/Matthias_Jakob_Schleiden) (Figure S1)

This cost-effective and culture-independent bioassay strictly adheres to [ISO Standard 20227](https://www.iso.org/standard/67326.html) [25].

*Spirodela polyrhiza* is an aquatic plant*, Angiosperm, Monocot, Alismatales, Araceae* from the genus *Spirodela.* It is a species of duckweed, a perennial, floating aquatic plant commonly found in freshwater bodies. While it is commonly used in wastewater bioremediation and foraging, its sensitivity to various chemicals allows its use as a toxicological bioindicator [82].

It can be found nearly worldwide in many types of freshwater habitat [83].

In aquatic ecosystems there are only a few standard methods to assess the hazard of toxicants to higher plants. Before the standardization in 2015 of the duckweed *S. polyrhiza* bioassay, there was only the standard method with duckweeds *Lemna minor* or *Lemna gibba* - a floating aquatic macrophyte in regular use in the EU for the authorization of Plant protection products (herbicides and plant regulators), but the *Lemna* bioassays are dependent on the culturing/maintenance of live stocks, they require substantial bench and incubation space, and they are hence quite costly.

DUCKWEED F microbiotest has been developed by selection of *S. polyrhiza* which is one of the very few duckweed species which produce “dormant vegetative buds (turions)”. Extensive experimental work has been performed on the biological and technical aspects for the production of turions of this species and on the storage medium and storage conditions for subsequent successful germination. A methodology was then developed for a “stock culture free” microbiotest with *S. polyrhiza*, departing from stored and germinated turions.


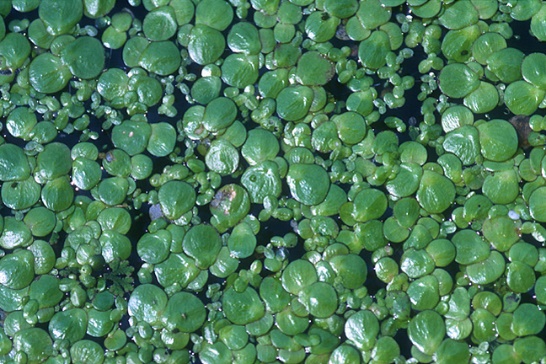


**Fig****ure S1.** *Spirodela polyrhiza* [84]

**The standard operating procedure steps**:

1. Preparation of duckweed growth and test dilution medium (Steinberg solution).
2. Germination of the *Spirodela polyrhiza* turions (incubation 72 hours at 25^0^C, under continuous illumination, at min. 6000 lux).
3. Preparation of the toxicant dilutions.


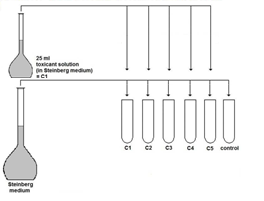


**Figure S2.** Operating scheme of Duckweed Toxkit F: preparing the toxicant dilution series [81].

1. Filling of the test plate with the toxicant dilutions.


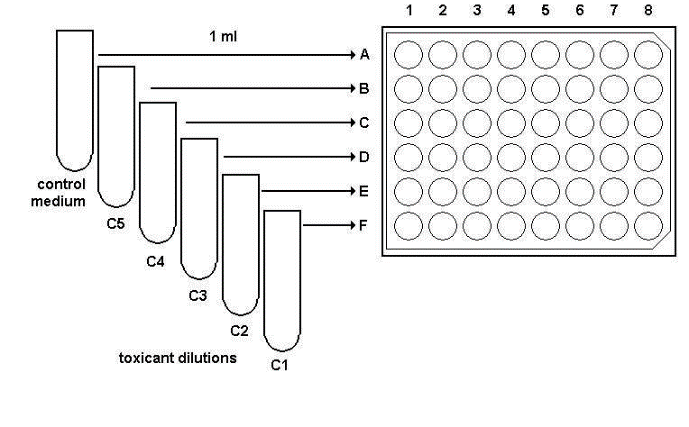

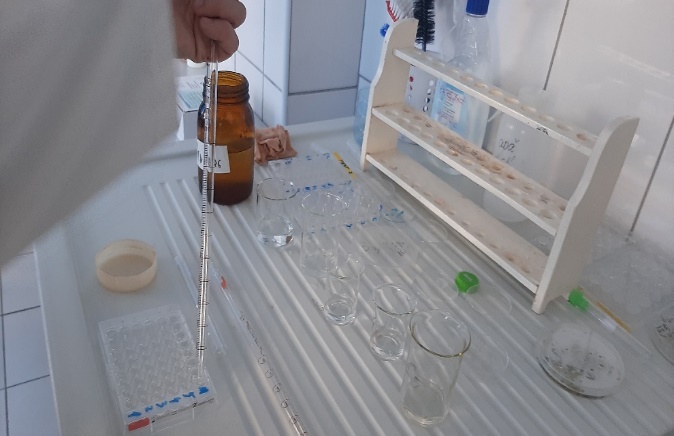


**Figure S3.** Filling of the test plate with the toxicant (TEB) dilution series and control medium, 8 wells/ for each variant of concentration ([81] and original photo).

1. Transfer of the germinated turions in the test cups, taking of a photo of the multiwell at the start of the toxicity test (original).


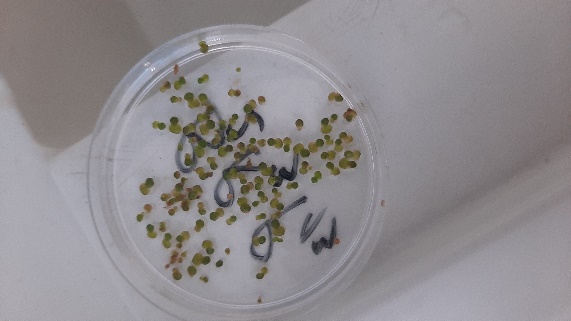

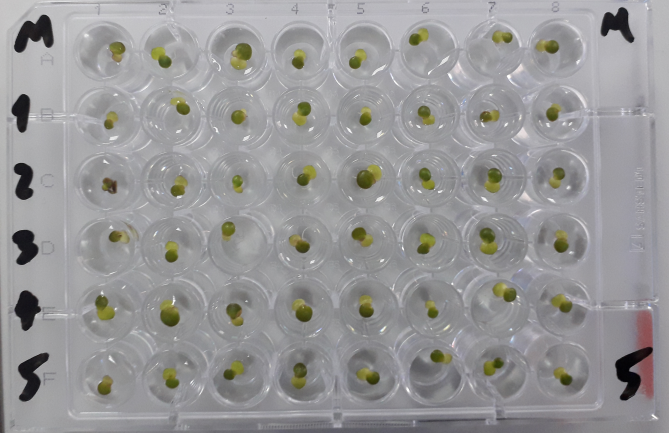


**Figure S4.** Transfer of the germinated turions in the test cups (original photos).

6. Incubation of the test plate (72 hours at 25^0^C, at min. 6000 lux).

7. Taking of a photo of the multiwell at the end of the toxicity test (Figure S5):


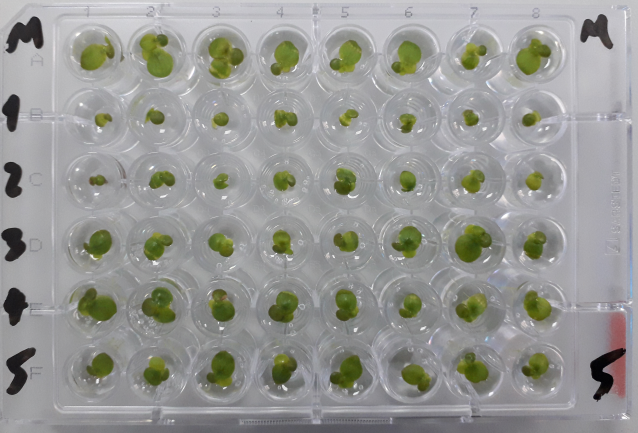


**Figure S5.** *S. polyrhiza* plants before after 72 hours after incubation in TEB-supplemented growth medium (M=control, 1-5=TEB concentrations) (original photos).

8. Measurement of the area of the first fronds, using the Image J sofware.

9*.* Validity of the test: The “mean growth” of the first fronds in the cups of the control column after 3 days incubation at 25 °C and under 6 000 lux illumination (= the mean t 72h – t 0h area) must be at least 10 mm^2^.

**DAPHTOXKIT F** magna with *Daphnia magna* Straus, 1820 (Figure S6)*.*

*D. magna is* a typical water flea of the genus *Daphnia -* Arthropoda, Crustacea, Branchiopoda, Anomopoda.

The acute *Daphnia magna* toxicity test is intended for toxicity screening of chemicals, effluents, surface waters, wastewaters, groundwaters, sediment pore waters and elutriates.

Daphtoxkit F contains all necessary materials to perform six acute 24-48h mobility inhibition tests with the freshwater crustacean *Daphnia magna*. The *Daphnia* immobilisation test is cost-effective, culture-independent, user-friendly and highly standardised, compliant to [ISO Standard 6341](https://www.iso.org/standard/54614.html) [28] and [OECD Guideline 202](https://www.oecd-ilibrary.org/environment/test-no-202-daphnia-sp-acute-immobilisation-test_9789264069947-en) [29].


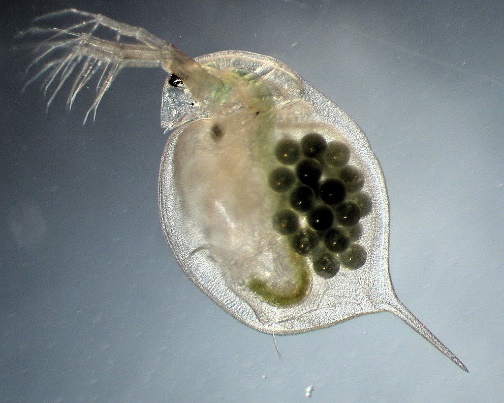
 **a**
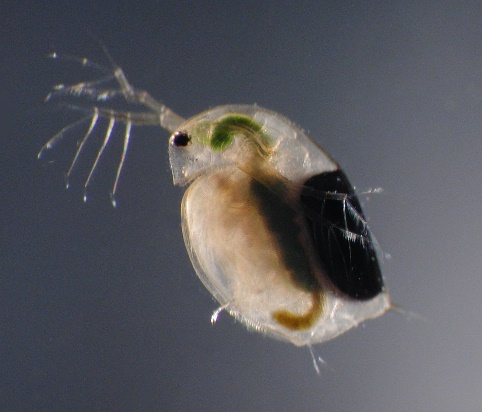
 **b**

**Figure S6.** *Daphnia magna*: female, with a clutch of eggs (a), carring an ephippium (b) [85,86].

**The standard operating procedure steps**:

- **Preparation of standard freshwater (S.F)**, used as hatching medium for the ephippia and as dilution medium for preparation of the toxicant dilution series.
- **Pre-aeration of S.F.** for at least 15 minutes prior to use it for the hatching of the dormant eggs and for the preparation of the toxicant dilution series.
- **Hatching of the ephippia** in diluted S.F., with 3 days prior to the start of the toxicity test, at 20-22^0^C, under continuous illumination, of min. 6000 lux.
- **Preparation of the toxicant dilution series** on chemical compounds, with two phases:

**● Range finding test (RFT)**, with a dilution series: 100 mg/L; 10 mg/L; 1 mg/L; 0,1 mg/L and 0,01 mg/L.

The result of RFT on TEB spanned one order of magnitude (case A from the table below): at a dilution of 10 mg/L there was 100% mortality and at a dilution of 1 mg/L there was 0% mortality.

Following the procedure, for the efinitive test (DT), C1 will be prepared according to the dilution instructions given in the table below:

| % mortality  …..100………….100 ------ 0………… ..0…………..  …..* ……………* ----------* …………..*…………..  C_1_ ….. C_5_ | Case A  one order of magnitude |
| --- | --- |
| % mortality  . . 100… 100 ----- 0 … 0…………..  …..* ….……* ---------* ---------*………*……  C_1_ C_5_ | Case B  two orders of magnitude |

**● Definitive test (DT).** The dilution series spans the range of the lowest concentration producing 100% mortality and the highest concentration producing 0% mortality in RFT with one order of magnitude. This concentration range is called C_1_ - C_5_.

The calculation of the actual concentrations of C_1_, C_2_, C_3_, C_4_ and C_5_, needed for the LC_50_ estimation is given below:

**C_1_ = 8.33 mg/L** (lowest concentration producing 100% mortality in RFT)

C_2_= 0.56 x C1= 4.66 mg/L

C_3_= 0.32 x C1= 2.66 mg/L

C_4_= 0.18 x C1= 1.49 mg/L

**C_5_= 0.1 x C1= 0.83 mg/L**

- **Filling of the test plate with the toxicant (TEB) dilution series and SF for control**, in 4 replicates (A, B, C and D) for each test variant.
- **Pre-feeding of the test organisms two hours prior of the start of exposure, with a suspension of *Spirulina* powder into SF** (Figure S7).


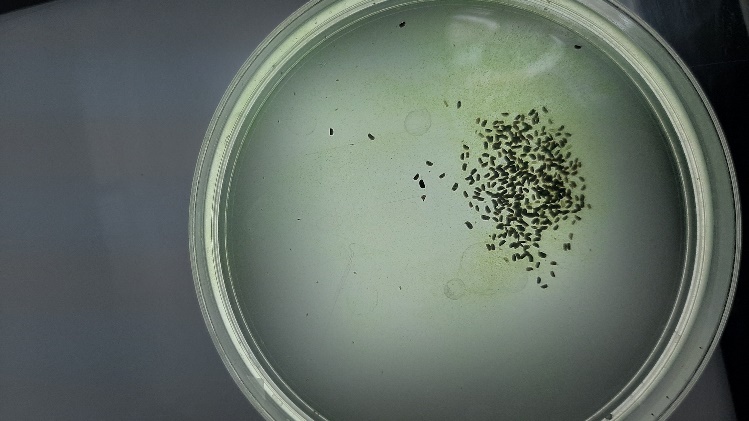

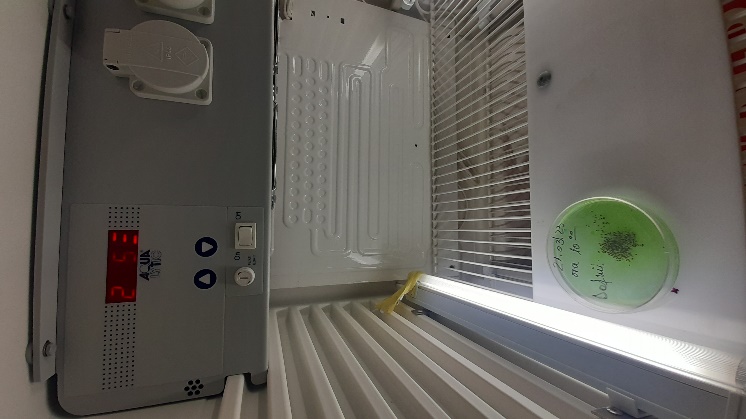


**Figure S7.** Pre-feeding the test organism with *Spirulina* powder.

- **Transfer of the neonates to the test wells** (5 *Daphnia* neonates in each well, with a total of 20 neonates for each test variant) (Figure S8).


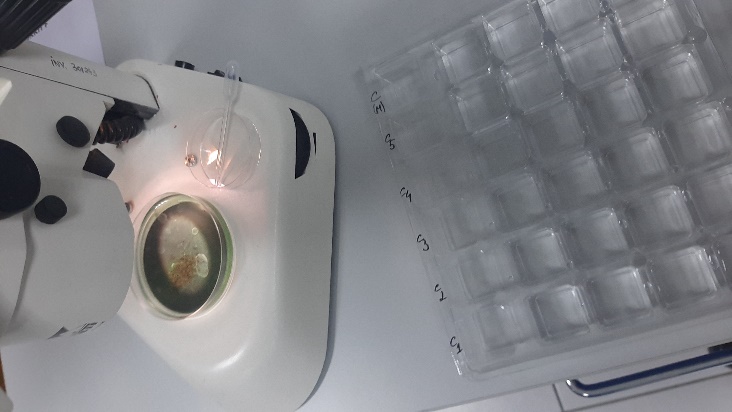


**Figure S8.** Transfer of the neonates in the test wells.

- **Incubation of the test plate:** the covered multiwell plate is incubated for 24 hours and 48 hours at 20^0^C in darkness.
- **Scoring of the results.** After 24 hours and 48 hours incubation the plate is placed under a dissection microscope and the dead and immobilized neonates (considered dead if they do not show any movement during 15 sec. of observation) are scored.
- **Estimation of the EC_50_ at 24 hours and 48 hours.** A data treatment program - based on Macro "REGTOX" (available on request from MicroBioTests Inc.) was applied using a sigmoid function with the EC_50_ calculation application (Table S1)

**Table S1.** Results after 48 h of exposure.

| **TEB**  **Concentration**  **mg/L** | | **Average effect** | | **Standard deviation** | **Nb replicates** | **R1** | **R2** | **R3** | **R4** |  |  |
| --- | --- | --- | --- | --- | --- | --- | --- | --- | --- | --- | --- |
| 0 | | 0 | | 0 | 4 | 0 | 0 | 0 | 0 |  |  |
| 0.83 | | 0.25 | | 0.5 | 4 | 0 | 1 | 0 | 0 |  |  |
| 1.49 | | 1.25 | | 0.5 | 4 | 2 | 1 | 1 | 1 |  |  |
| 2.66 | | 3 | | 0 | 4 | 3 | 3 | 3 | 3 |  |  |
| 4.66 | | 4.25 | | 0.5 | 4 | 4 | 4 | 4 | 5 |  |  |
| 8.33 | | 5 | | 0 | 4 | 5 | 5 | 5 | 5 |  |  |
| **Calc. Parameters** |  | | **Parameters values** | |  | **Confidence intervals** | |  | |  | **500 Set of simulated Ys, replicates** |
| **HILL** | **Optimal** | | **Average** | | **Median** | **< alpha =5** | | **> alpha =5** | | **< alpha =1** | **> alpha =1** |
|  | 0 | | No object : non ajusted parameter | | | | |  | |  |  |
|  | 2.502 | | 2.527 | | 2.523 | 1.908 | | 3.197 | | 1.716 | 3.279 |
|  | 2.370 | | 2.382 | | 2.376 | 2.231 | | 2.564 | | 2.154 | 2.634 |
|  | 5.153 | | 5.170 | | 5.153 | 4.967 | | 5.468 | | 4.967 | 5.591 |
| EC_5_ | 0.730 | | 0.736 | | 0.749 | 0.518 | | 0.941 | | 0.459 | 0.962 |
| EC_10_ | 0.985 | | 0.989 | | 0.992 | 0.783 | | 1.188 | | 0.708 | 1.208 |
| EC_15_ | 1.185 | | 1.189 | | 1.185 | 0.994 | | 1.374 | | 0.920 | 1.391 |
| EC_20_ | 1.362 | | 1,366 | | 1.375 | 1.170 | | 1.532 | | 1.110 | 1.547 |
| EC_50_ | 2.370 | | 2.382 | | 2.376 | 2.231 | | 2.564 | | 2.154 | 2.634 |

**THAMNOTOXKIT F with *Thamnocephalus platyurus*** (Figure S9)

***Thamnocephalus platyurus*** is a typical beaver-tail fairy shrimp of the genus *Thamnocephalus* -*Arthropoda, Crustacea, Branchiopoda, Anostraca, Thamnocephalus platyurus* Packard, 1877.

The cyst-based *Thamnocephalus platyurus* toxicity test is intended for toxicity screening of chemicals, effluents, biotoxins, surface waters, wastewaters, groundwaters, sediment pore waters and elutriates.


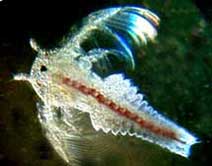


**Figure S9.** *Thamnocephalus platyurus* larva stage 2-3 [87]

Thamnotoxkit F contains all necessary materials to perform six acute 24 h mortality tests with the freshwater crustacean *T. platyurus*. This cost-effective, culture-independent and highly-standardised bioassay is compliant with [ISO Standard 14380](https://www.iso.org/standard/54613.html) [30].

**The standard operating procedure steps**:

- **Preparation of standard freshwater (S.F)**, according to the US EPA formula, used as hatching medium for the cysts and for the toxicant dilution series preparation.
- **Pre-aeration of S.F.** and storage of 1 L S.F. (6 bioassays of each Toxkit).
- **Hatching of the cysts** (Figure S10) - in diluted S.F., for 20-22 hours at 25^°^C, under continuous illumination, at min. 3000-4000 lux.


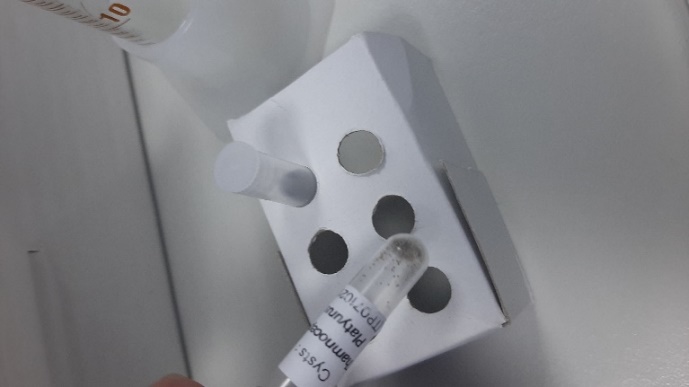


Figure S10. *Thamnocephalus platyurus* cysts.

- **Preparation of the toxicant dilution series** on chemical compounds, with two phases:

**● Range find test (****RFT)**, with a dilution series: 100 mg/L; 10 mg/L; 1 mg/L; 0,1 mg/L and 0,01 mg/L.

The result of RFT on TEB spanned two order of magnitude (case B from the table below): at a dilution of 1 mg/L there was 100% mortality and at a dilution of 0,01 mg/L there was 0% mortality.

Following the procedure, for the definitive test (DT), C1 will be prepared according to the dilution instructions given in the table below:

| % mortality  …..100………….100 ------ 0………… ..0…………..  …..* ……………* ----------* …………..*…………..  C_1_ ….. C_5_ | Case A  one order of magnitude |
| --- | --- |
| % mortality  . . 100… 100 ----- 0 … 0…………..  …..* ….……* ---------* ---------*………*……  C_1_ C_5_ | Case B  two orders of magnitude |

**● Definitive test.** The dilution series spans the range of the lowest concentration producing 100% mortality and the highest concentration producing 0% mortality in RFT with two orders of magnitude. This concentration range is called C_1_ - C_5_.

Calculation of the actual concentrations of C_1_, C_2_, C_3_, C_4_ and C_5_, needed for the LC_50_ estimation:

**C_1_ = 1 mg/L** (lowest concentration producing 100% mortality in RFT)

C_2_= 0.32 x C1= 0.32 mg/L

C_3_= 0.18 x C1= 0.18 mg/L

C_4_= 0.1 x C1= 0.1 mg/L

**C_5_= 0.03 x C1= 0.03 mg/L**

- **Filling of the test plate with the toxicant (TEB) dilution series and SF for control**, in 3 replicates (A, B and C) for each test variant.
- **Transfer of the larvae to the test wells** (10 larvae in each well, with a total of 30 larvae for each test variant) (Figure S11).


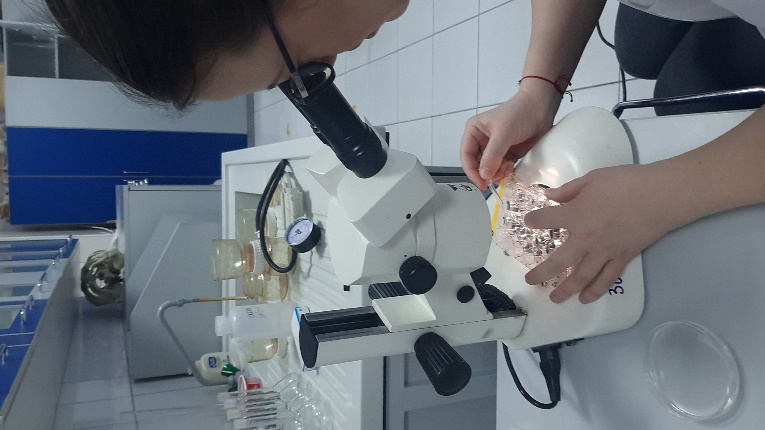


**Figure S11**. Transfer of the larvae to the test wells.

- **Incubation of the test plate:** the covered multiwell plate is incubated for 24 hours, at 25^°^C in darkness.
- **Scoring of the results.** Under a dissection microscope the mortality of the larvae is scored (considered dead if they do not show any movement during 10 sec. of observation).
- **Estimation of the LC_50_ at 24 hours.** A data treatment program - based on Macro "REGTOX" (available on request from MicroBioTests Inc.) was applied using a sigmoid function with the LC_50_ calculation application (Table S1).

**Table S2. Results after 24 hours of exposure.**

| **TEB**  **Concentration**  **Mg/L** | | **Average effect** | | **Standard deviation** | **Nb replicates** | | **R1** | | **R2** | | **R3** | |  |  |
| --- | --- | --- | --- | --- | --- | --- | --- | --- | --- | --- | --- | --- | --- | --- |
| 0 | | 0 | | 0 | 3 | | 0 | | 0 | | 0 | |  |  |
| 0.03 | | 0.67 | | 0,58 | 3 | | 1 | | 1 | | 0 | |  |  |
| 0.1 | | 3.33 | | 0.58 | 3 | | 3 | | 3 | | 4 | |  |  |
| 0.18 | | 9 | | 0 | 3 | | 9 | | 9 | | 9 | |  |  |
| 0.32 | | 10 | | 0 | 3 | | 10 | | 10 | | 10 | |  |  |
| 1 | | 10 | | 0 | 3 | | 10 | | 10 | | 10 | |  |  |
| **Calc. Parameters** |  | | **Parameters values** | | |  | | **Confidence intervals** | |  | |  | | **500 Set of simulated Ys, replicates** |
| **HILL** | **Optimal** | | **Average** | | | **Median** | | **< alpha =5** | | **> alpha =5** | | **< alpha =1** | | **> alpha =1** |
|  | 0 | | No object: non ajusted parameter | | | | | | |  | |  | |  |
|  | 4.817 | | 4.810 | | | 4.817 | | 4.234 | | 5.128 | | 4.136 | | 5.148 |
|  | 0.115 | | 0.115 | | | 0.115 | | 0.110 | | 0.118 | | 0.110 | | 0.118 |
|  | 10.043 | | 10.044 | | | 10.043 | | 10.031 | | 10.069 | | 10.028 | | 10.088 |
| EC5 | 0,063 | | 0.063 | | | 0.063 | | 0.055 | | 0.066 | | 0.054 | | 0.067 |
| EC10 | 0.073 | | 0.073 | | | 0.073 | | 0.065 | | 0.077 | | 0.065 | | 0.077 |
| EC15 | 0.081 | | 0.080 | | | 0.081 | | 0.073 | | 0.084 | | 0.072 | | 0.084 |
| EC20 | 0.087 | | 0.086 | | | 0.087 | | 0.079 | | 0.090 | | 0.079 | | 0.090 |
| EC50 | 0.115 | | 0.115 | | | 0.115 | | 0.110 | | 0.118 | | 0.110 | | 0.118 |

**References:**

81. Toxkit microbiotests procedures – Available online: http://www.microbiotests.com (accessed on 23 March 2023)

82. Baudo, R.; Foudoulakis, M.; Arapis, G.; Perdaen, K.; Lanneau, W.; Paxinou, A.C.M.; Kouvdou, S.; Persoone, G. History and sensitivity comparison of the *Spirodela polyrhiza* microbiotest and *Lemna* toxicity tests. *Knowl. Manag. Aquat. Ecosyst.* 2015, *416*, doi: 10.1051/kmae/2015019.

83. Wang, Wenqin; Kerstetter, Randall A.; Michael, Todd P. “Evolution of Genome Size in Duckweeds (Lemnaceae)”, Journal of Botany. 2011:1-9. Available online: <https://doi.org/10.1155/2011/570319>, ISSN 2090-0120 (accessed on 10.04.2023)

84. Image of *Spirodela polyrhiza (Spirodela polyrrhiza* marais poitevin - Wikipedia), (accessed in 14.04.2023)

85. Image of *Daphnia magna* a: Available online: https://www.google.ro/url?sa=i&url=https%3A%2F%2Fen.wikipedia.org%2Fwiki%2FDaphnia_magna&psig=AOvVaw2bpxOLMn59Qbpmzy7uo4RU&ust=1681554345020000&source=images&cd=vfe&ved=0CBEQjRxqFwoTCMiJrZ6Uqf4CFQAAAAAdAAAAABAQ accessed on 14.04.2023.

86. Image of Daphnia magna b: Available online: https://www.google.ro/url?sa=i&url=https%3A%2F%2Fen.wikipedia.org%2Fwiki%2FDaphnia_magna&psig=AOvVaw2bpxOLMn59Qbpmzy7uo4RU&ust=1681554345020000&source=images&cd=vfe&ved=0CBEQjRxqFwoTCMiJrZ6Uqf4CFQAAAAAdAAAAABAI (accessed on 14.04.2023)

87. Image of *Thamnocephalus platyurus* - Thamnocephalus platyurus Available online: https://www.biotoxicity.com (accessed on 14.04.2023)
