# Supplementary material for: Multiple Assays on Non-Target Organisms to Determine the Risk of Acute Environmental Toxicity in Tebuconazole-Based Fungicides Widely Used in the Black Sea Coastal Area
Source: Toxics. 2023 Jul 7;11(7):597. doi: 10.3390/toxics11070597 (PMC10385278; doi:10.3390/toxics11070597)
Supplement: Supplementary file 1 [file toxics-11-00597-s001.zip › S3. Experiments on bacteria and yeasts.docx]

**S3. Experiments on bacteria and yeasts**

| 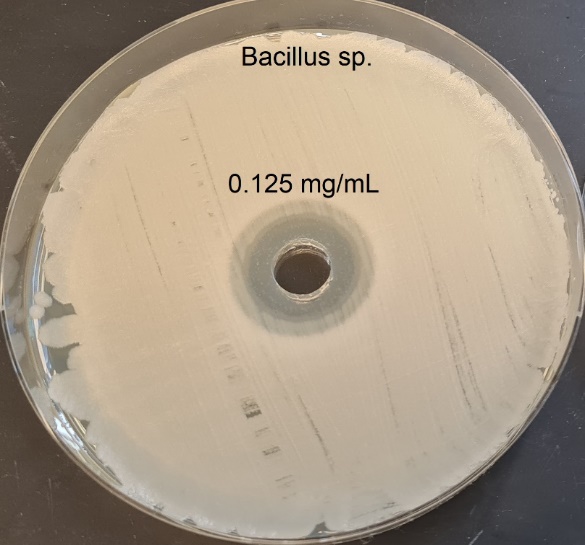**A** | 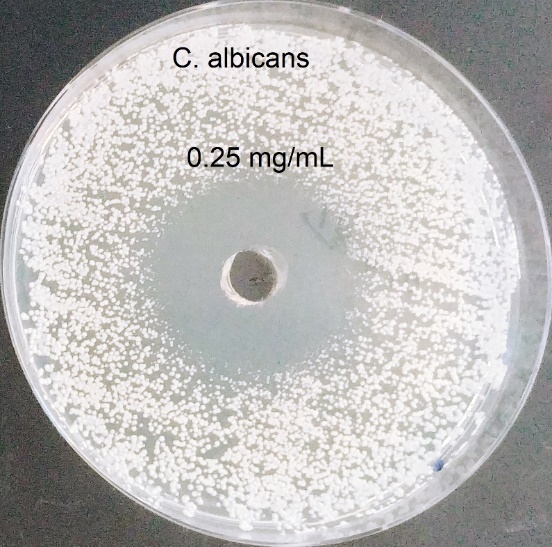**B** |
| --- | --- |
| 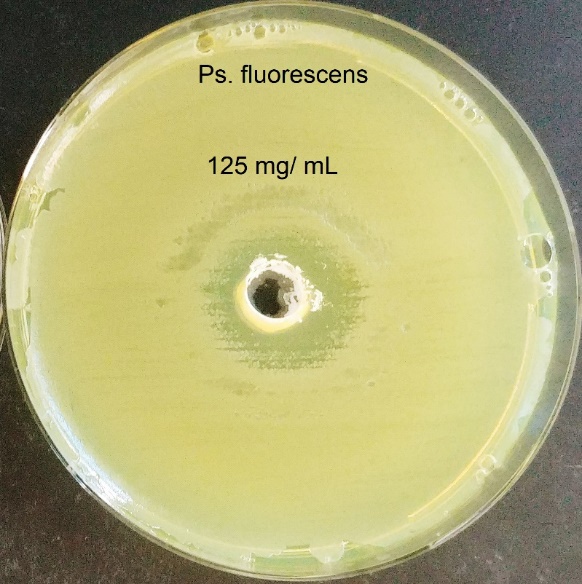**C** | 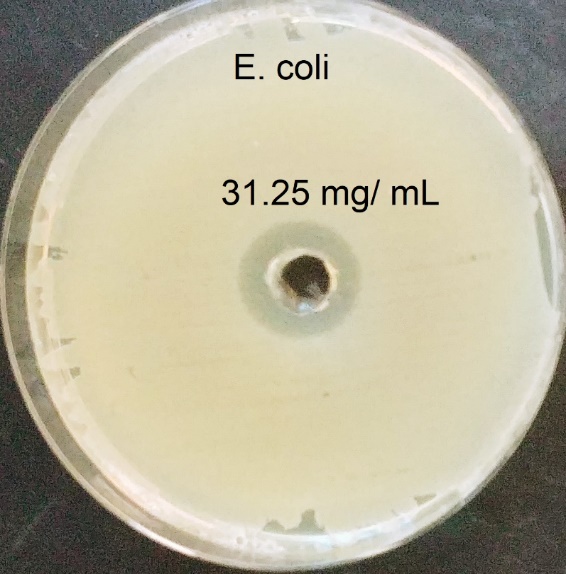**D** |
| 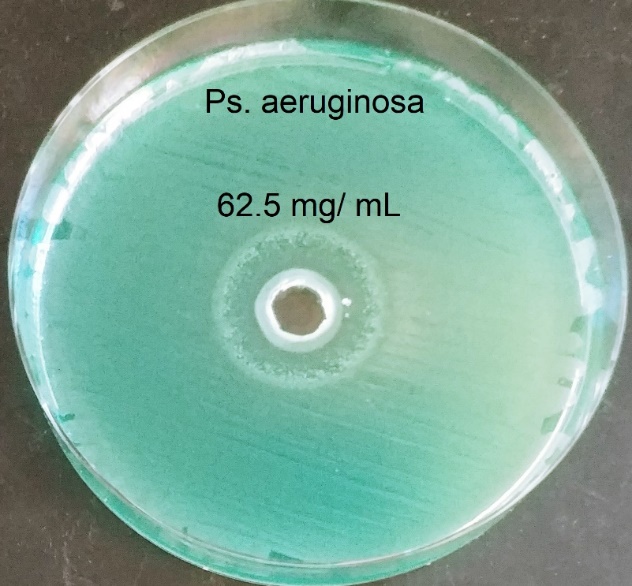**E** | |

**Figure S1.** Susceptibility of microorganisms to tebuconazole. The toxic effect of tebuconazole as growth inhibition zones in diffusimetric tests; *Bacillus sp*. (A), *Candida albicans* ATCC 10231 (B), *Pseudomonas fluorescens (C), Escherichia coli* ATCC 25922 (D), *Pseudomonas aeruginosa* ATCC 27853 (E).
