# Supplementary material for: Multiple Assays on Non-Target Organisms to Determine the Risk of Acute Environmental Toxicity in Tebuconazole-Based Fungicides Widely Used in the Black Sea Coastal Area
Source: Toxics. 2023 Jul 7;11(7):597. doi: 10.3390/toxics11070597 (PMC10385278; doi:10.3390/toxics11070597)
Supplement: Supplementary file 1 [file toxics-11-00597-s001.zip › S4. Analysis of water samples for pesticide content detection.docx]

**S4. Analysis of water samples for pesticide content detection**

The interest of the academic and scientific environment is particularly high regarding the development of a green agriculture in Dobrogea, a county located in the east of Romania, between the Danube River and the Black Sea, where agricultural productions are mostly represented by wheat, barley, rapeseed, vineyards and fruit trees (Figures S1 & S2). Constanta is a city with more than 264,000 inhabitants and the main source of drinking water for the population is the Danube River - Black Sea canal, which crosses many agricultural lands where fungicide/pesticide treatments are applied.

Through our studies we want to make known the serious environmental effects of pesticides and to influence decision makers to monitor pesticides and their residues in ground and surface water resources. Limiting the use of highly toxic pesticides will protect aquatic ecosystems and human health dependent on water quality.


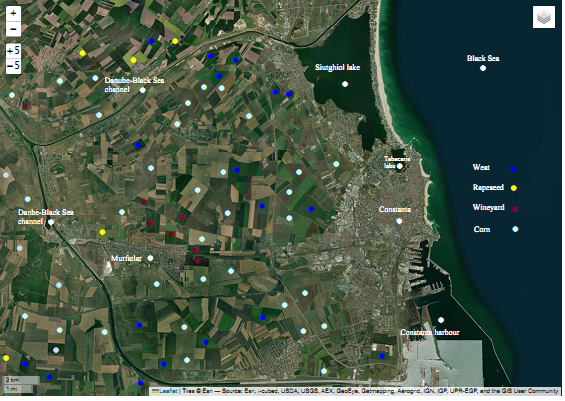


**Figure S1.** Map of agricultural land along Romanian Black Sea Coast, where TEBUSTAR EW fungicide treatments are applied.


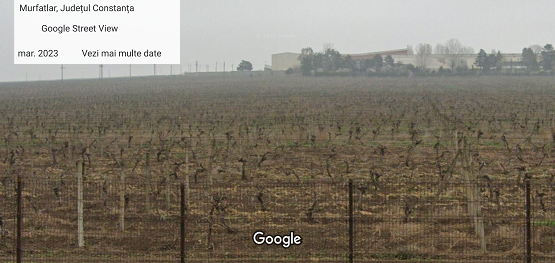


**Figure S2.** Murfatlar vineyard situated along the canall connecting the Danube River with Black Sea

**Water sampling from the Tăbăcărie and Siutghiol lakes, to analyze pesticide content:**

Tăbăcărie L. (Figures S1, S3) is an urban lake, located in the northern part of Constanta. The lake is surrounded by various commercial units (City Mall, tourist units and restaurants), social and cultural units (Children's Park, Summer Theatre and an Orthodox settlement) and a Wastewater Treatment Plant. If 40 years ago the vegetation area around the lake was dominant and the lake water was relatively clean, in the last three decades the vegetation has been replaced by numerous socio-economic objectives and the lake has become a polluted ecosystem, characterized in the summer season by hyper-eutrophication and loss of biodiversity. The risk of contamination with various toxic substances has increased and it will be necessary to take measures to rehabilitate the ecosystem in order not to endanger the existence of aquatic organisms (plants, invertebrates, fish and birds) (Figures S3, S4). The danger is high due to the practice of fishing in this lake, without fishermen knowing the risk of fish consumption.


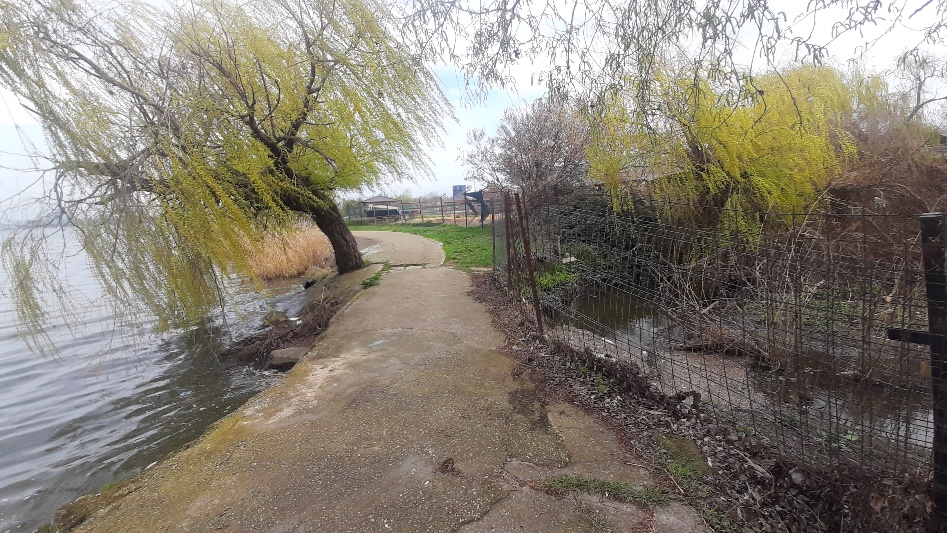

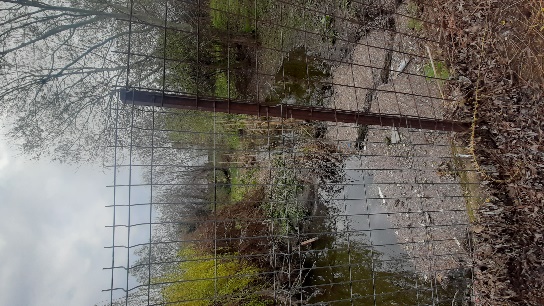


**Figure S3.** Tăbăcărie Lake - a canal discharging polluted waters into the lake.


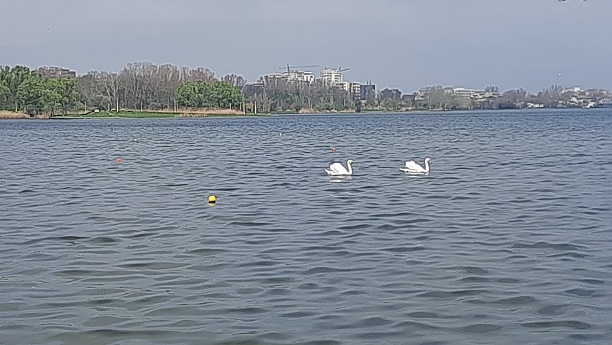

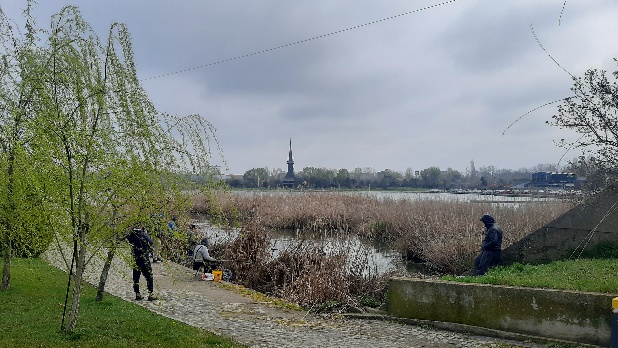


**Figure S4.** Migratory birds on Tăbăcărie lake (left). Fishing activities in the shallow waters.

Lake Siutghiol (Figure S5) communicates with Lake Tăbăcărie, and Lake Tăbăcărie flows into the Black Sea, its waters flowing directly onto the beach in the north of Constanta, marking the boundary between the city and the tourist resort of Mamaia, which is situated in the north of Constanta, on a strip of sandy coastline framed to the east by the Black Sea and to the west by Lake Siutghiol (also called Mamaia, after the name of the resort) (see Figure S1). In recent decades, Lake Siutghiol has also suffered the impact of urban development (new residential areas surrounding the lake), to the detriment of maintaining the vegetation. Being a lake with a much larger surface area than Lake Tăbăcărie, there are still areas of land that are used for local agriculture. The water quality of this lake is affected in the summer season when the anthropic impact increases due to tourist and recreational activities on the lake.


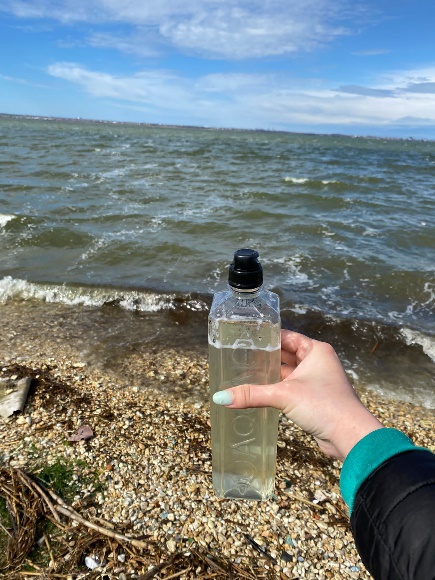

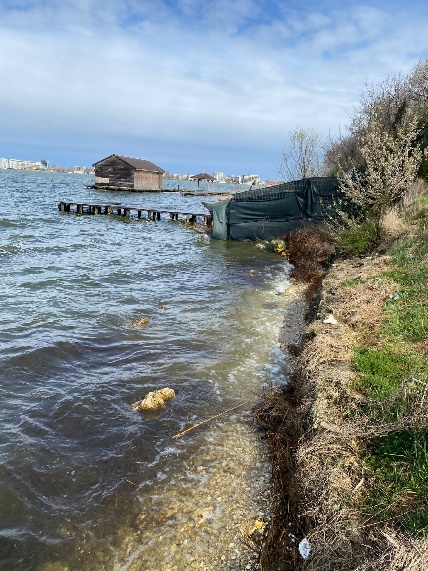

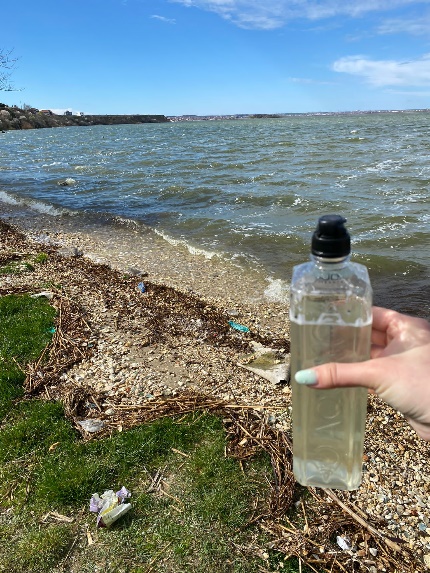


**Figure S5.** Siutghiol Lake - locations of water sampling for Tebuconazole detection.
