# Supplementary material for: Multiple Assays on Non-Target Organisms to Determine the Risk of Acute Environmental Toxicity in Tebuconazole-Based Fungicides Widely Used in the Black Sea Coastal Area
Source: Toxics. 2023 Jul 7;11(7):597. doi: 10.3390/toxics11070597 (PMC10385278; doi:10.3390/toxics11070597)
Supplement: Supplementary file 1 [file toxics-11-00597-s001.zip › S5. Duckweektoxkit F Spirodela Regtox statistics.pdf]

Bootstrap calculation of toxicity parameters
11.05.2023 15:32
Data sheet : Spirodela Regtox

Concentration	Average effect	Standard deviation	Nb replicates								
0	11,5905	4,290565198	8	14,047	16,453	4,011	14,954	10,827	13,084	6,491	12,857
0,39	9,774	3,533733032	8	10,057	5,06	16,272	6,081	11,539	8,835	11,744	8,604
0,781	9,03025	3,989371048	8	15,787	2,15	9,487	7,351	8,187	7,315	9,549	12,416
1,562	8,247625	4,8693659	8	14,29	8,873	2,628	3,056	5,395	12,422	14,186	5,131
3,125	2,228125	1,61848884	8	1,245	1,792	0,696	3,036	0,107	5	3,639	2,31
6,25	0,96775	0,884383522	8	2,58	0,714	0,133	0,55	0,185	0,475	2	1,105

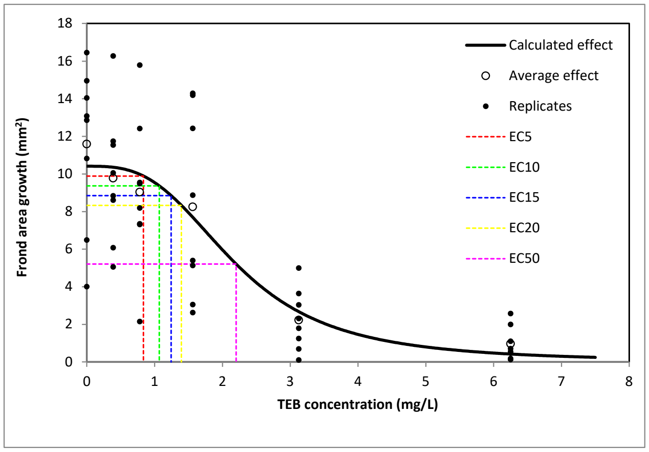

Calc. Parameters	Parameters values			Confidence intervals		500 Set of simulated Ys, replicates	
HILL	Optimal	Average	Median	< alpha =5	> alpha =5	< alpha =1	> alpha =1
Control	10,41010258	10,92598169	10,77590656	8,972294331	13,6716671	8,456084251	14,1005373
Hill number	3,035215132	4,704181911	2,785853148	1,042898834	19,37869072	0,901856303	21,10332203
EC50	2,203653411	2,043036499	2,068352699	1,030846894	2,946077585	0,790752381	2,98356843
Maximum effect	0	No object : non ajusted parameter					
EC5	0,835290489	0,904797242	0,710850954	0,070255131	2,503999114	0,039089581	2,571987391
EC10	1,068446763	1,077408187	0,936161339	0,140314221	2,610549808	0,086428419	2,662812114
EC15	1,24436414	1,21194613	1,108632922	0,212706208	2,674214363	0,139664613	2,720815063
EC20	1,395677349	1,331400085	1,268330574	0,293511704	2,727120161	0,196252652	2,766692042
EC50	2,203653411	2,043036499	2,068352699	1,030846894	2,946077585	0,790752381	2,98356843
