# Supplementary material for: Multiple Assays on Non-Target Organisms to Determine the Risk of Acute Environmental Toxicity in Tebuconazole-Based Fungicides Widely Used in the Black Sea Coastal Area
Source: Toxics. 2023 Jul 7;11(7):597. doi: 10.3390/toxics11070597 (PMC10385278; doi:10.3390/toxics11070597)
Supplement: Supplementary file 1 [file toxics-11-00597-s001.zip › S6. MBT Daphnia Regtox statistics.pdf]

Bootstrap calculation of toxicity parameters
11.05.2023 19:11 Dafnia EC50 effect 48h exposure
Data sheet : Daphnia Regtox 48 h

| Concentration | Average effect | Standard deviation | Nb replicates | | | | |
|---|---|---|---|---|---|---|---|
| 0 | 0 | 0 | 4 | 0 | 0 | 0 | 0 |
| 0,83 | 0,25 | 0,5 | 4 | 0 | 1 | 0 | 0 |
| 1,49 | 1,25 | 0,5 | 4 | 2 | 1 | 1 | 1 |
| 2,66 | 3 | 0 | 4 | 3 | 3 | 3 | 3 |
| 4,66 | 4,25 | 0,5 | 4 | 4 | 4 | 4 | 5 |
| 8,33 | 5 | 0 | 4 | 5 | 5 | 5 | 5 |

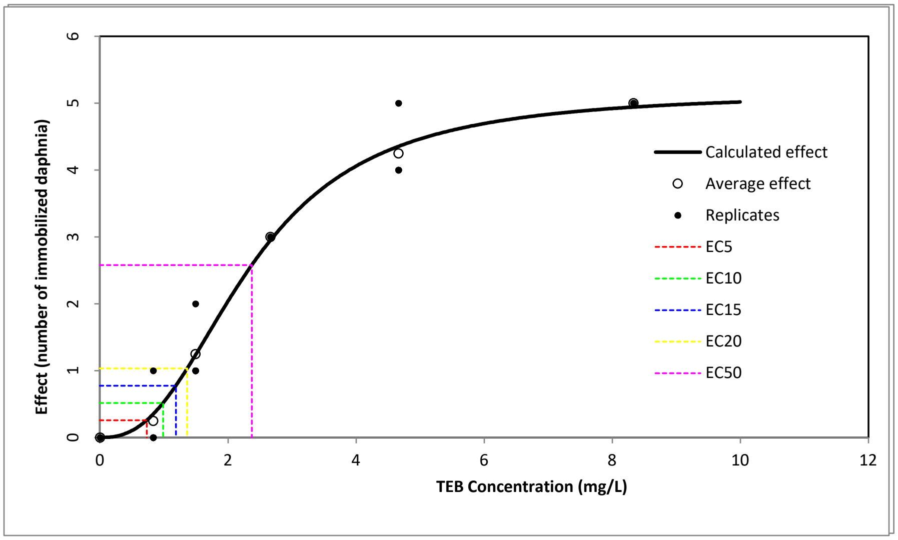


| Calc. Parameters | Parameters values | | | Confidence intervals | | 500 Set of simulated Ys, replicates | |
|---|---|---|---|---|---|---|---|
| HILL | Optimal | Average | Median | < alpha =5 | > alpha =5 | < alpha =1 | > alpha =1 |
| | | 0 No object : non ajusted parameter | | | | | |
| | 2,502947519 | 2,504007362 | 2,502947569 | 1,819105864 | 3,022921085 | 1,616360247 | 3,197910309 |
| | 2,370349588 | 2,386764714 | 2,376816988 | 2,266146183 | 2,521183968 | 2,200393558 | 2,730052471 |
| | 5,153528455 | 5,171792055 | 5,153528214 | 4,967662334 | 5,48139596 | 4,967662334 | 5,766492367 |
| EC5 | 0,730993166 | 0,729466136 | 0,730993152 | 0,511837989 | 0,897031665 | 0,440301031 | 0,941230357 |
| EC10 | 0,985291857 | 0,983267539 | 0,985291839 | 0,780392498 | 1,148828506 | 0,700545162 | 1,188972831 |
| EC15 | 1,185321585 | 1,183789867 | 1,185321569 | 0,98646912 | 1,338995814 | 0,922812402 | 1,374038219 |
| EC20 | 1,362297641 | 1,361843916 | 1,362297654 | 1,174956799 | 1,502674103 | 1,118208289 | 1,53214848 |
| EC50 | 2,370349588 | 2,386764714 | 2,376816988 | 2,266146183 | 2,521183968 | 2,200393558 | 2,730052471 |
