# Supplementary material for: Multiple Assays on Non-Target Organisms to Determine the Risk of Acute Environmental Toxicity in Tebuconazole-Based Fungicides Widely Used in the Black Sea Coastal Area
Source: Toxics. 2023 Jul 7;11(7):597. doi: 10.3390/toxics11070597 (PMC10385278; doi:10.3390/toxics11070597)
Supplement: Supplementary file 1 [file toxics-11-00597-s001.zip › S7. MBT Thamno Regtox statistics.pdf]

Bootstrap calculation of toxicity parameters
11.05.2023 19:59 Thamno TEB exposure 24 h
Data sheet : Thamno Regtox

Concentration	Average effect	Standard deviation	Nb replicates			
0	0	0	3	0	0	0
0,03	0,666666667	0,577350269	3	1	1	0
0,1	3,333333333	0,577350269	3	3	3	4
0,18	9	0	3	9	9	9
0,32	10	0	3	10	10	10
1	10	0	3	10	10	10

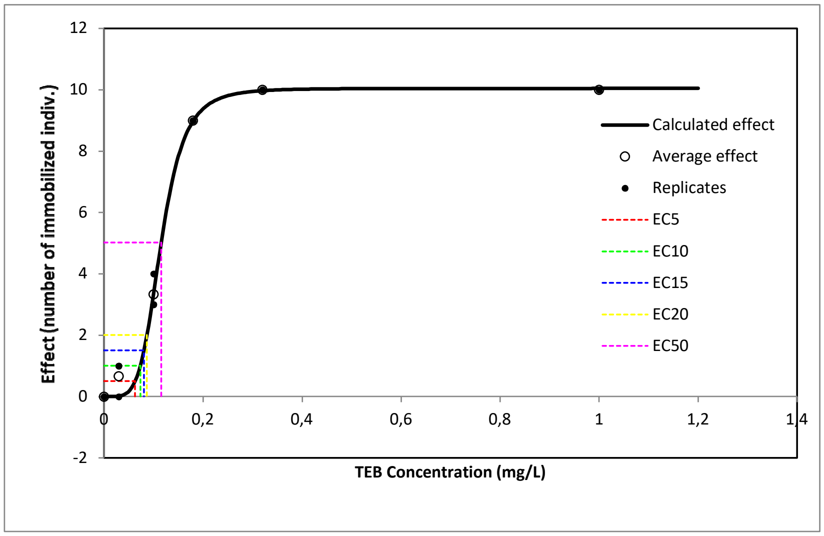

Calc. Parameters	Parameters values		Confidence intervals		500 Set of simulated Ys, replicates		
HILL	Optimal	Average	Median	< alpha =5	> alpha =5	< alpha =1	> alpha =1
Control	0	No object : non ajusted parameter					
Hill number	4,817441048	4,812290209	4,817445755	4,233829498	5,127745152	4,135730267	5,148203373
EC50	0,115493338	0,115415424	0,115493305	0,110020727	0,118115485	0,109943286	0,118115485
Maximum effect	10,04267707	10,04382865	10,04267502	10,03081894	10,0694437	10,02762032	10,08755493
EC5	0,062677899	0,062564443	0,06267792	0,054883543	0,06646885	0,054021135	0,066597596
EC10	0,073194112	0,073076273	0,073194124	0,065477081	0,07689596	0,064718574	0,077000298
EC15	0,080571578	0,080454254	0,080571584	0,073037177	0,084156066	0,072378442	0,084240049
EC20	0,086612759	0,086497975	0,086612761	0,079299837	0,090071052	0,078738116	0,09013661
EC50	0,115493338	0,115415424	0,115493305	0,110020727	0,118115485	0,109943286	0,118115485
