# Supplementary figures and images for: Multiple Assays on Non-Target Organisms to Determine the Risk of Acute Environmental Toxicity in Tebuconazole-Based Fungicides Widely Used in the Black Sea Coastal Area
Source: Toxics. 2023 Jul 7;11(7):597. doi: 10.3390/toxics11070597 (PMC10385278; doi:10.3390/toxics11070597)

**S2. Experiments on marine fish (*Chelon auratus*)**


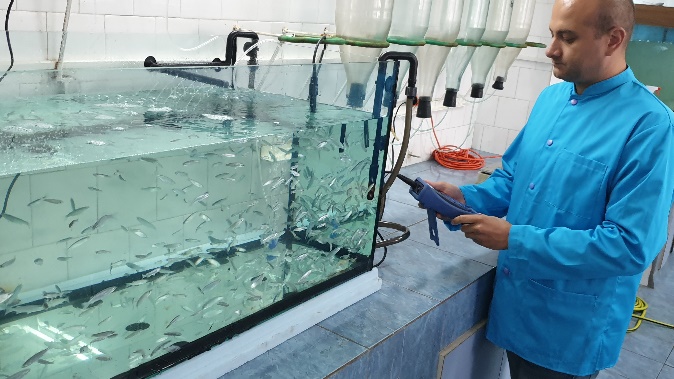


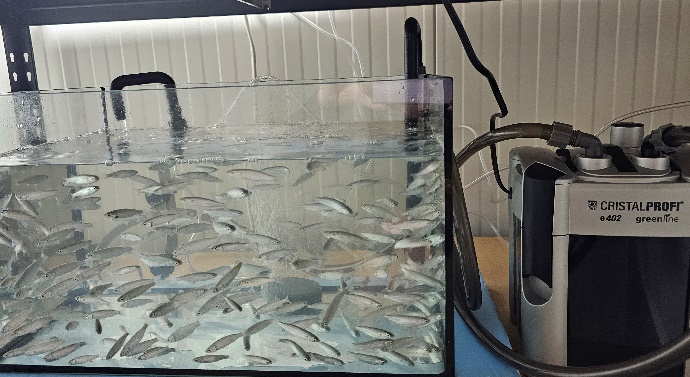


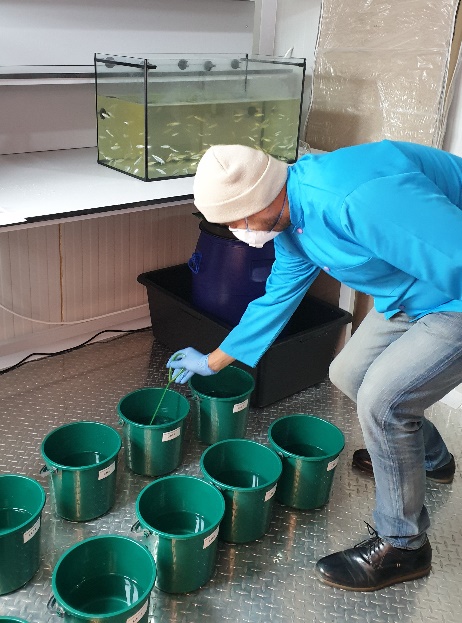

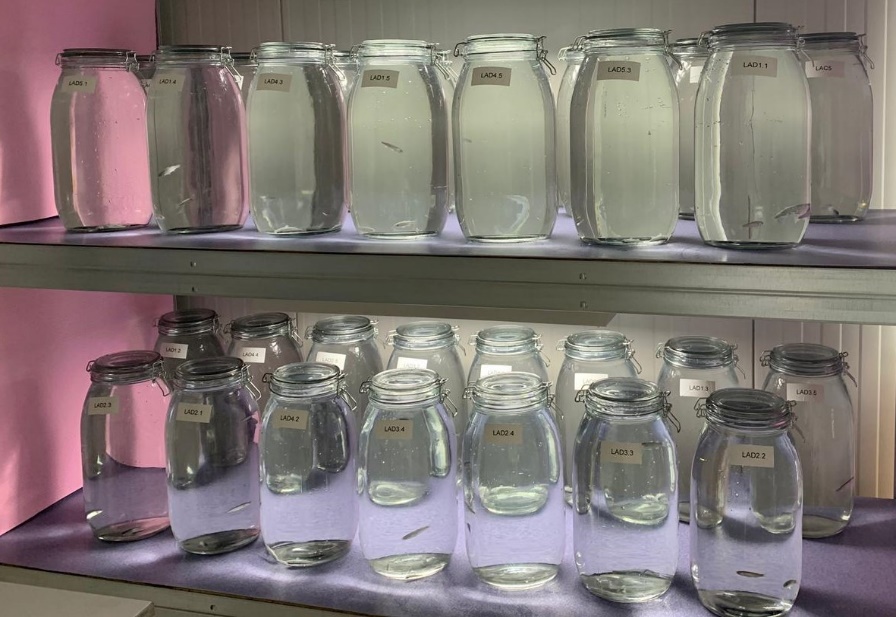


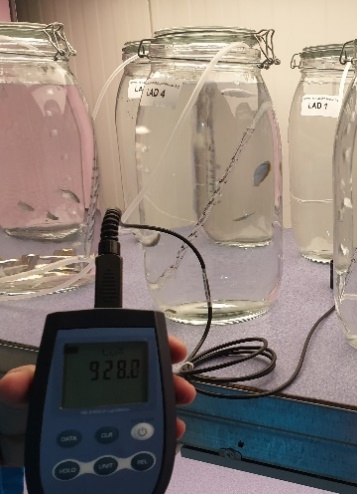

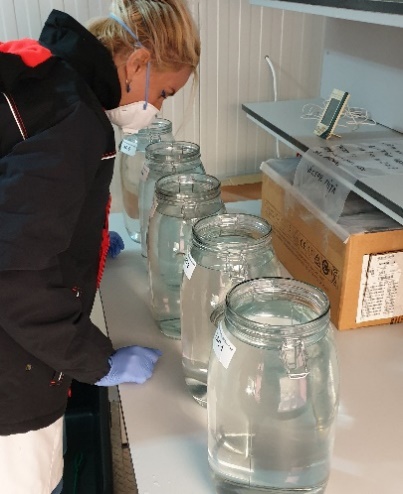

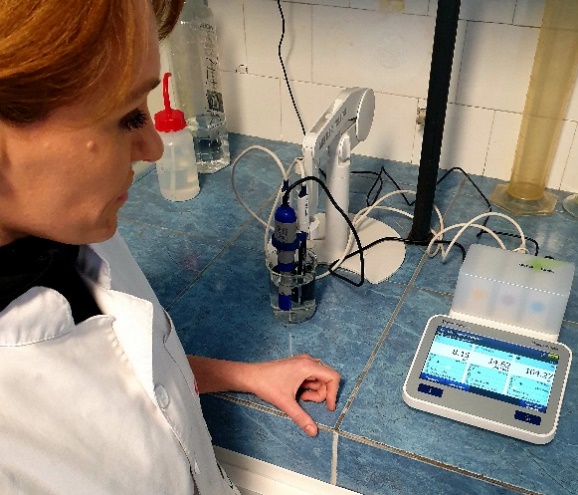

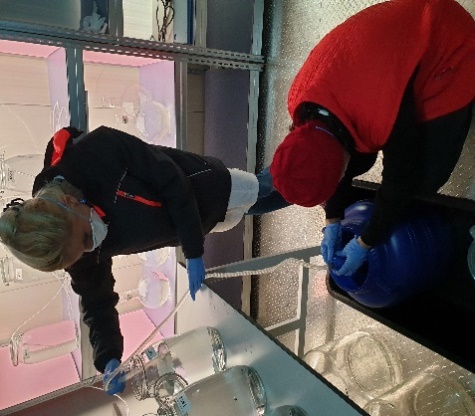


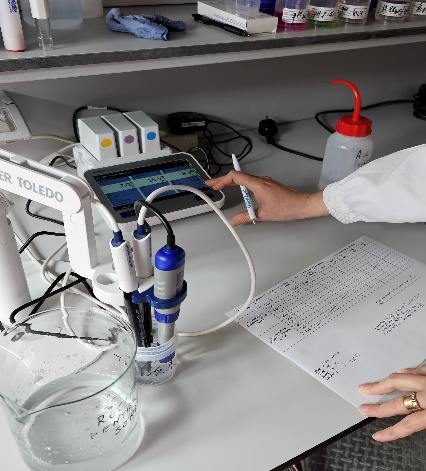

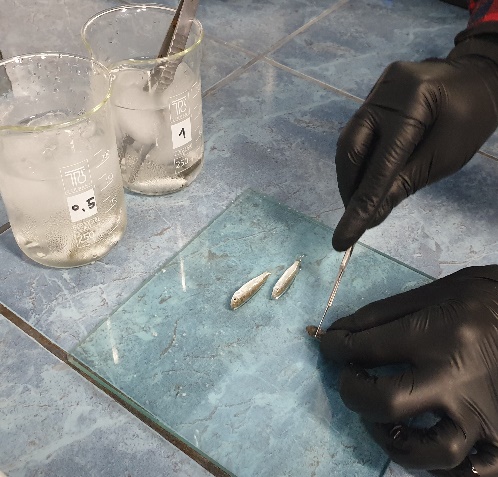

Supplement: Supplementary file 1 [file toxics-11-00597-s001.zip › S2. Experiments on marine fish (Chelon auratus).docx]
